# Neuroligin-3 and neuroligin-4X form nanoscopic clusters and regulate growth cone organization and size

**DOI:** 10.1093/hmg/ddab277

**Published:** 2021-09-20

**Authors:** Nicholas J F Gatford, P J Michael Deans, Rodrigo R R Duarte, George Chennell, Katherine J Sellers, Pooja Raval, Deepak P Srivastava

**Affiliations:** Department of Basic and Clinical Neuroscience, Institute of Psychiatry, Psychology, & Neuroscience, King's College London, London, UK; MRC Centre for Neurodevelopmental Disorders, Institute of Psychiatry, Psychology and Neuroscience, King's College London, London, UK; Department of Basic and Clinical Neuroscience, Institute of Psychiatry, Psychology, & Neuroscience, King's College London, London, UK; MRC Centre for Neurodevelopmental Disorders, Institute of Psychiatry, Psychology and Neuroscience, King's College London, London, UK; Department of Basic and Clinical Neuroscience, Institute of Psychiatry, Psychology, & Neuroscience, King's College London, London, UK; Department of Basic and Clinical Neuroscience, Institute of Psychiatry, Psychology, & Neuroscience, King's College London, London, UK; Department of Basic and Clinical Neuroscience, Institute of Psychiatry, Psychology, & Neuroscience, King's College London, London, UK; Department of Basic and Clinical Neuroscience, Institute of Psychiatry, Psychology, & Neuroscience, King's College London, London, UK; MRC Centre for Neurodevelopmental Disorders, Institute of Psychiatry, Psychology and Neuroscience, King's College London, London, UK; Department of Basic and Clinical Neuroscience, Institute of Psychiatry, Psychology, & Neuroscience, King's College London, London, UK; MRC Centre for Neurodevelopmental Disorders, Institute of Psychiatry, Psychology and Neuroscience, King's College London, London, UK

## Abstract

The cell-adhesion proteins neuroligin-3 and neuroligin-4X (NLGN3/4X) have well described roles in synapse formation. NLGN3/4X are also expressed highly during neurodevelopment. However, the role these proteins play during this period is unknown. Here we show that NLGN3/4X localized to the leading edge of growth cones where it promoted neuritogenesis in immature human neurons. Super-resolution microscopy revealed that NLGN3/4X clustering induced growth cone enlargement and influenced actin filament organization. Critically, these morphological effects were not induced by autism spectrum disorder (ASD)-associated NLGN3/4X variants. Finally, actin regulators p21-activated kinase 1 and cofilin were found to be activated by NLGN3/4X and involved in mediating the effects of these adhesion proteins on actin filaments, growth cones and neuritogenesis. These data reveal a novel role for NLGN3 and NLGN4X in the development of neuronal architecture, which may be altered in the presence of ASD-associated variants.

## Introduction

In mature neurons, axo-dendritic structure is an essential aspect of neuronal function as this is a defining component of neural network connectivity (1). Development of axo-dendritic morphology begins immediately after neural commitment with the formation and extension of protrusions (neurites) from the cell soma (2). This process is referred to as neuritogenesis. During this process, immature neurites with actin-rich growth cones form as neural progenitor cells differentiate. Subsequent differentiation leads to the development of the axon and primary dendrite. Extrinsic cues and intrinsic mechanisms work in parallel to orchestrate the development of neuronal morphology (3). Neuritogenesis is driven by growth cones, which are highly dynamic and motile subcellular structures (1–4). Growth cone behaviour depends on filamentous actin (F-actin) and microtubule dynamics and is regulated by complex signalling pathways (1,2,5). A major regulator of actin that is also involved in regulating growth cone dynamics is p21-activated kinase (PAK1) (2,6). PAK1, in concert with other actin regulators, enables actin treadmilling and ensures a balance between stable and unstable F-actin in the growth cone. This actin treadmilling provides the growth cone with significant flexibility allowing exploration of the extracellular matrix, response to chemotropic cues and communication with adjacent cells via cell-adhesion (4).

Cell-adhesion molecules (CAMs) are also critical regulators of this signalling pathway, operating as molecular clutches between cells which modulate growth cone stabilization or destabilization based on cell–cell or cell-matrix interactions (1,2,7,8). Accumulating evidence suggests that the neurexin-neuroligin cell-adhesion complex (NRXN-NLGN) regulates axonal development (9–11). NLGNs are a family of post-synaptic proteins which bind to pre-synaptic NRXN; enabling trans-synaptic adhesion, synapse maturation and establishing synaptic identity in an activity-dependent manner (12–14). To date, most studies examining NLGN function have focused on mature neurons, particularly at synapses. Little attention has been given to examining the role of the NLGNs during early neuronal development, despite clear developmental expression of NLGNs (10,15,16). However, there are some key exceptions. NLGN1 and NRXN have been found to contribute to dendritogenesis in Xenopus neurodevelopment (17). Furthermore, NLGN1 clusters at axonal branch points and filopodia tips during Drosophila cellular neurodevelopment where it acts as a stabilizer, ultimately contributing to axonal arborisation (9). These studies provide substantial insight into the role of NLGN1 in neurodevelopment but do not examine other NLGNs. Interestingly, multiple gene mutations in the NRXN-NLGN cell-adhesion complex are strongly associated with neurodevelopmental disorders, particularly autism spectrum disorders (ASD); the pathogenesis of which is currently associated with mutations in over 1000 genes (13,18,19). The contribution of such mutations to atypical neurodevelopment has been highlighted by recent studies using patient-derived induced pluripotent stem cell models (20,21). In the NRXN-NLGN cell-adhesion complex, two key mutations in NLGN3 and NLGN4X. NLGN3-R451C is caused by a single nucleotide polymorphism (SNP) resulting in an arginine to cysteine amino acid change in the acetylcholinesterase domain. NLGN4X-D396 is caused by a novel stop codon at position 396 resulting in a premature truncation of the NLGN4X protein (22,23). These have been well described to impact synapse structure as well as function (24). However, the role of NLGN3/4X in neurodevelopment and whether the ASD-associated mutations of these proteins impact neurodevelopment and/or the development of neuronal architecture is not known.

In this study, we demonstrate a novel role for NLGN3/4X during early human neurodevelopment. Using a conditionally immortalized human neural progenitor cell line, we show that immature neurons endogenously express NLGN3/4X, and that these adhesion proteins form nanoscopic clusters at the leading edge of growth cones. These clusters appear to increase substantially when NLGN3/4X are ectopically expressed. ASD-associated mutant variants of these proteins display an altered nanoscopic distribution. Ectopic NLGN3/4X expression promoted neurite outgrowth, coupled with an enlargement of growth cone size. Using super-resolution imaging, we observed actin filaments reorganized both with regards to structural organization as well as bundling, as growth cones enlarged. Interestingly, NLGN3/4X ASD mutants did not exert these effects on neurites or growth cones. We further show that PAK1 was required for both NLGN3/4X-mediated growth cone enlargement as well as nanoscopic clustering of NLGN3/4X. Combined, these results suggest that the CAM NLGN3/4X regulate growth cone structure and organization via modulation of actin, ultimately promoting neuritogenesis in human neurodevelopment. Critically, the morphological effects on immature neurons induced by NLGN3 and NLGN4X were not recapitulated by ASD-associated mutants. This may suggest that alterations in growth cone organization and neuritogenesis may contribute to ASD pathophysiology.

## Results

### NLGN3 and NLGN4X induce neurite outgrowth in primary cortical neurons

**Figure 1:**
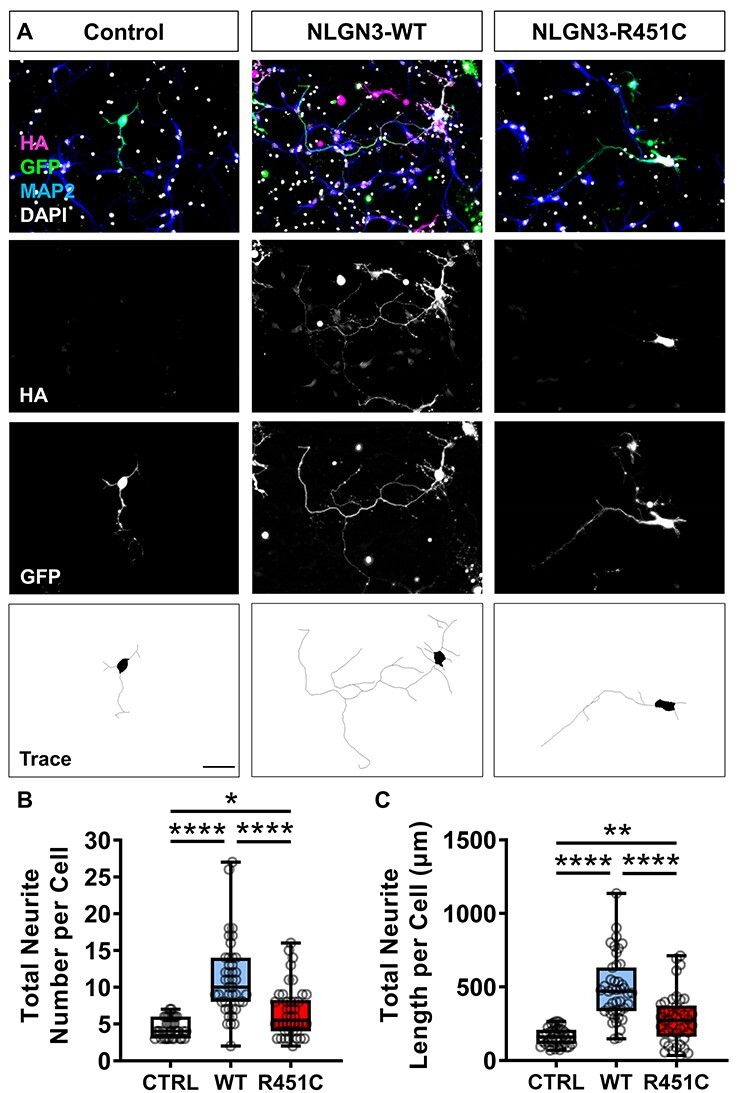
NLGN3-WT and NLGN3-R451C increase neuritogenesis in mouse primary cortical neurons. (A) Representative images showing ectopic NLGN3-WT and NLGN3-R451C expression significantly increases neurite number and length in mouse primary cortical neurons. Scale bar = 50 μm. (B) Box plots of data demonstrating NLGN3-WT and NLGN3-R451C expression increases neurite number confirmed by parametric one-way ANOVA with Bonferroni post-hoc correction or non-parametric Kruskal–Wallis *H* test with Dunn’s post-hoc test. (C) Box plots of data demonstrating NLGN3-WT and NLGN3-R451C expression increases neurite length confirmed by parametric one-way ANOVA with Bonferroni post-hoc correction or non-parametric Kruskal–Wallis *H* test with Dunn’s post-hoc test.

**Figure 2:**
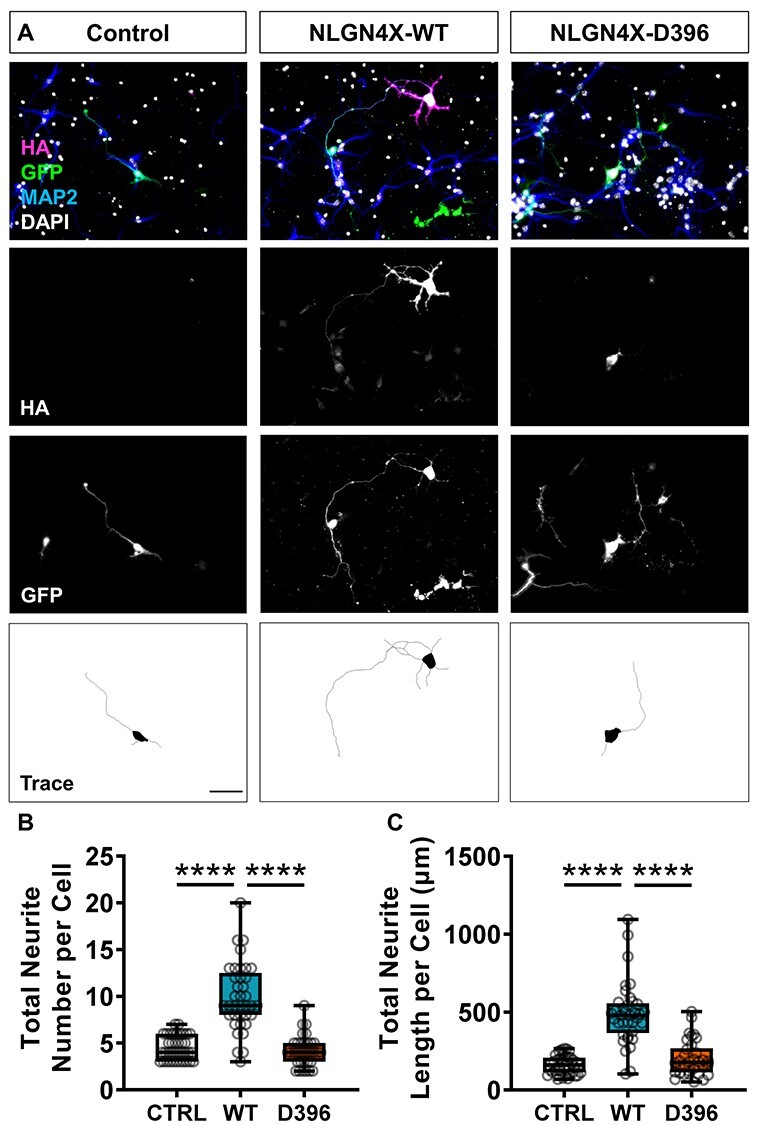
NLGN4X-WT but not NLGN4X-D396 increase neuritogenesis in mouse primary cortical neurons. (A) Representative images showing ectopic NLGN4X-WT expression significantly increases neurite number and length in mouse primary cortical neurons. Scale bar = 50 μm. (B) Box plots of data demonstrating NLGN4X-WT expression increases neurite number confirmed by parametric one-way ANOVA with Bonferroni post-hoc correction or non-parametric Kruskal–Wallis *H* test with Dunn’s post-hoc test. (C) Box plots of data demonstrating NLGN4X-WT expression increases neurite length confirmed by parametric one-way ANOVA with Bonferroni post-hoc correction or non-parametric Kruskal–Wallis *H* test with Dunn’s post-hoc test.

Neuroligin-1 (NLGN1) and neurexin contribute to dendritogenesis in Xenopus neurodevelopment (17). To assess whether a similar effect could be observed in a mammalian system, we ectopically expressed HA-tagged wildtype (WT) NLGN3, NLGN4X or the ASD-associated mutant variants HA-NLGN3-R451C or HA-NLGN4X-D396 in developing mouse cortical neurons. Significant increases in neurite outgrowth were observed in NLGN3/4X-WT transfected DIV4 (days in vitro) mouse cortical neurons compared to GFP-transfected controls (Figs 1A and 2A). This increase was not found in the mutant conditions, although NLGN3-R451C significantly increased neurite outgrowth but not to the extent seen in the NLGN3-WT condition (Figs 1B and 2B). By DIV4, mouse cortical neurons have established a single axonal projection, defined as the longest neurite, and several dendrite protrusions emerging from the soma (25). Therefore, we assessed whether NLGN3/4X-WT expression was preferentially regulating either compartment. Ectopic expression of either NLGN3-WT or NGN4X-WT increased both axon and dendrite number and length (Supplemental Material, Fig S1A–D). Axon and dendrite growth were increased to a similar level by NLGN3-WT and NGN4X-WT **(**Supplemental Material, Fig S1E–F). Ectopic expression of NLGN3-R451C only increased axon length, whereas NLGN4X-D396 did not alter axon or dendrite growth **(**Supplemental Material, Fig S1A–D). Taken together, these data support a role for NLGN3 and NLGN4X in the development of neuronal morphology in a mammalian system.

### NLGN3 and NLGN4X are expressed in early human neurodevelopment

**Figure 3:**
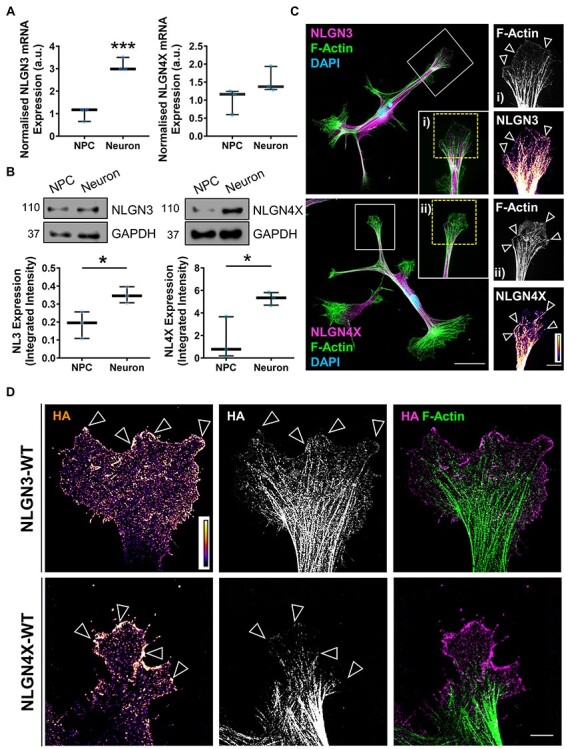
NLGN3 and NLGN4X are expressed in developing human neurons and form nanoscopic growth cones clusters. (A) RT-qPCR data showing endogenous NLGN3 and NLGN4X mRNA expression levels increase as hNPCs differentiate into immature neurons, confirmed by *t*-test (*n* = 3). (B) Data and representative western blots showing endogenous NLGN3 and NLGN4X protein expression levels increase as hNPCs differentiate into immature neurons, confirmed by *t*-test (*n* = 3). (C) Representative super-resolution images of immature human neurons showing endogenous NLGN3 (upper) NLGN4X (lower) expression and localisation. NLGN3 and NLGN4X localize to the growth cone, particularly at the leading edge where they colocalise with F-actin (insets). Scale bar = 25 μm (whole cell), 5 μm (insets). (D) Representative super-resolution images of human neuronal growth cones ectopically expressing HA-tagged NLGN3 or NLGN4X. Clusters of HA-NLGN3/4X are visible at the growth cone leading edge based on high intensity puncta (open white arrows). Scale bar = 5 μm.

The NLGNs, and their extracellular binding partner NRXNs, are expressed in the mature rodent brain and during rodent and chick embryonic development (15,16). However, the temporal expression pattern of NLGN3/4X in human neurodevelopment is unknown. Compiled data from the BrainSpan Atlas of the Developing Human Brain (26) revealed NLGN3 and NLGN4X are expressed in the human prenatal brain throughout neurodevelopment and NLGN3 is expressed almost twice as highly as NLGN4X **(**Supplemental Material, Fig S2**)**. To confirm NLGN3/4X expression in immature human neurons, we assessed NLGN3/4X mRNA expression in undifferentiated human neural progenitor cells (hNPCs) and immature neurons. NLGN3 exhibited a 3.34 fold-change increase in endogenous mRNA expression between neural progenitor and immature neuron stages. NLGN4X exhibited a 1.467 fold-change increase in endogenous mRNA expression between neural progenitor and immature neuron stages **(**Fig 3A**)**. Similar to mRNA expression, NLGN3 and NLGN4X protein levels significantly increased as cells adopted a neuronal fate **(**Fig 1B**;****Fig S3****and****S4****)**. This supports the BrainSpan data indicating NLGN3/4X are expressed during the prenatal period, and therefore, may have a functional role during this stage of neurodevelopment.

### NLGN3 and NLGN4X localize to the leading edge of growth cones

To gain insight into the role of NLGN3/4X in early neurodevelopment, we first assessed subcellular distribution of endogenous NLGN3/4X in immature neurons by super-resolution microscopy. NLGN3 and NLGN4X were expressed diffusely throughout the cell. However, discrete NLGN3/4X puncta could be identified along neurites as well as at the leading edge of growth cones where NLGN3/4X colocalized with actin filaments **(**Fig 3C**)**. Previous studies have shown that recruitment and subsequent clustering of NLGNs to synapses is critical for their ability to influence synaptic structure and function (27–29). Thus, we reasoned NLGN3/4X would also cluster during neurodevelopment. To promote clustering, we ectopically expressed HA-NLGN3-WT or HA-NLGN4X-WT in differentiating neurons for 3 days. Both proteins were expressed throughout the cell **(**Supplemental Material, **Fig S5****)**. However, both proteins were particularly enriched in growth cones, where they could be observed as nanoscopic clusters at the growth cone leading edge **(**Fig 3D**)**. Taken together, these data indicate NLGN3 and NLGN4X are expressed at growth cones in immature neurons and, upon clustering, enrich at the leading edge of growth cones.

### Mutant NLGN3 and NLGN4X display abnormal subcellular distributions

Previous studies have demonstrated correct subcellular localization of NLGN3/4X is critical for their ability to influence synaptic structure and function (30–32). Interestingly, ASD-associated NLGN3/4X mutants have been shown to mislocalize in mature neurons. NLGN3-R451C and NLGN4X-D396, for example, are retained intracellularly (22,23). To investigate whether this mislocalization was recapitulated in our cellular system, we compared subcellular localization of ectopic NLGN3/4X-WT and its mutant forms in hNPCs. HA-NLGN3-R451C was predominately localized within the cytosol, with only a fraction of the protein at the plasma membrane **(**Supplemental Material, **Fig S6****)**. Similarly, HA-NLGN4X-D396 was almost completely localized intracellularly in agreement with previous reports **(**Supplemental Material, **Fig S6****).**

**Figure 4:**
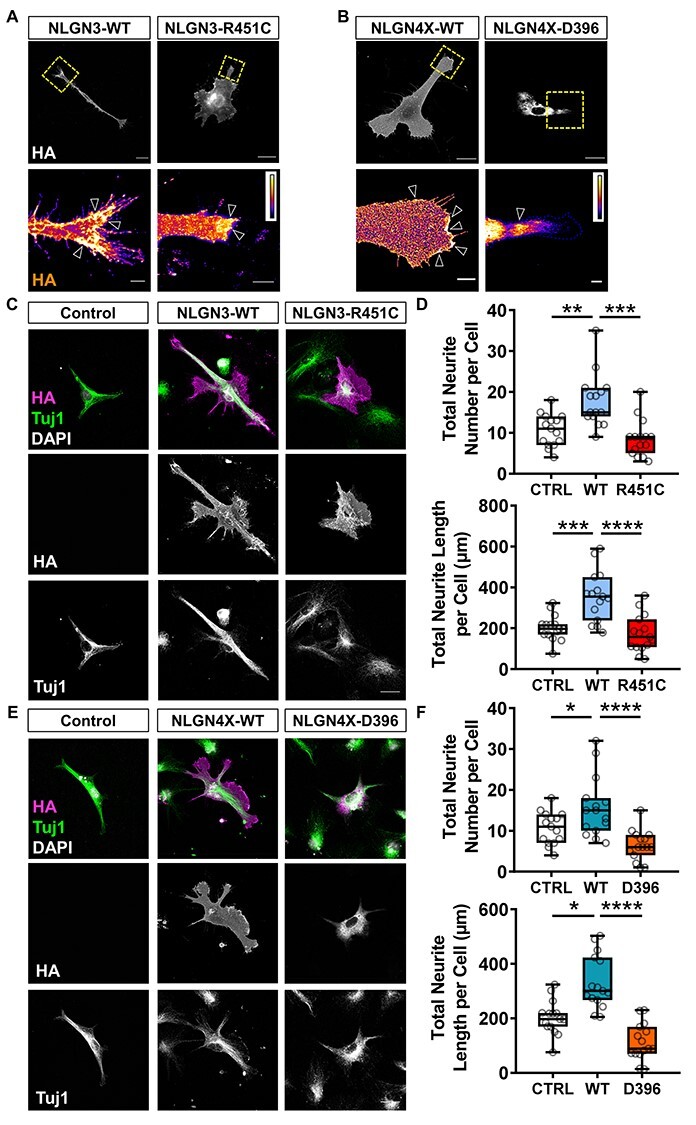
NLGN3/4X-WT but not ASD-associated variants increase neuritogenesis in developing human neurons. (A) Representative intensity images showing ectopic NLGN3-WT is highly localized to the leading edge of growth cones while the mutant variant is less localized but still present at the leading edge (open white arrows). Scale bar = 5 μm. (B) Representative intensity images showing ectopic NLGN4X-WT is highly localized to the leading edge of growth cones while NLGN4X-D396 is barely present in growth cones (open white arrows). Scale bar = 5 μm. (C + D) Representative images and data showing ectopic NLGN3 expression significantly increases neurite number and length in immature neurons, confirmed by parametric one-way ANOVA with Bonferroni post-hoc correction or non-parametric Kruskal–Wallis *H* test with Dunn’s post-hoc test (*n* = 15). Scale bar = 25 μm. (E + F) Representative images and data showing ectopic NLGN4X expression significantly increases neurite number and length in immature neurons, confirmed by parametric one-way ANOVA with Bonferroni post-hoc correction or non-parametric Kruskal–Wallis *H* test with Dunn’s post-hoc test (*n* = 15). Scale bar = 25 μm.

As our data indicated that NLGN3/4X-WT were particularly enriched at growth cones in immature neurons **(**Fig 3D**),** we next compared the distribution of NLGN3/4X-WT and their mutant variants in immature neurons **(**Fig 4A and B**)**. Examination of growth cones of immature neurons revealed that NLGN3-R451C was less present at the leading edge but less abundant than the WT form **(**Fig 4A**)**. Conversely, the NLGN4X-D396 mutant was found to aggregate within the neurite and not localize to the plasma membrane, unlike NLGN4X-WT which localized to the leading edge of growth cones **(**Fig 4B**)**. Taken together, these data indicate that ASD-associated mutant NLGN3 and NLGN4X display reduced presence at the plasma membranes of hNPCs and abnormal localization at the leading edge of growth cones in immature neurons.

### NLGN3-WT, NLGN4X-WT and their mutant forms exert differential effects on neurite outgrowth

NLGN3/4X have well established roles in synaptogenesis and spine formation, an effect mediated by the clustering of these proteins at synaptic sites which regulates actin cytoskeletal remodeling (27,31,33,34). We, therefore, reasoned that as WT NLGN3 and NLGN4X clustered at membranes of hNPCs and at the leading edge of growth cones in immature neurons, that these adhesion proteins may be involved in regulating membrane structures and neurite outgrowth. Indeed, ectopic NLGN3 expression in hNPCs significantly increased cell membrane features not induced by NLGN3-R451C. This could be demonstrated by increased lamellipodia on protrusions, which is indicative of cytoskeletal reorganization at the plasma membrane **(**Supplemental Material, **Fig S7****A and B)**. Next, we examined whether NLGN3-WT or NLGN3-R451C could induce changes in neuritogenesis owing to the enrichment of this adhesion protein at growth cones. In line with the mouse data, ectopic NLGN3-WT expression significantly increased neurite count and neurite length in immature neurons **(**Fig 4C and D**)**. This was found to be driven by a significant increase in the number and length of secondary neurites **(**Supplemental Material, **Fig S7C****)**.

We then investigated whether NLGN4X may also influence cell membrane structures owing to its functional similarity to NLGN3. In hNPCs, ectopic NLGN4X-D396 expression altered cell membrane features compared to control. This was demonstrated by significant decreases in the number of concave membrane sections, lamellipodia and lamellipodia on protrusions compared to control **(**Supplemental Material, **Fig S7****A and B)**. This suggests the NLGN4X-D396 mutation may operate in a dominant negative way to negatively influence membrane structures.

Next, we ectopically expressed NLGN4X-WT in immature neurons to determine whether this protein also influenced neuritogenesis. Ectopic NLGN4X-WT expression also significantly increased neurite count and neurite length **(**Fig 4E and F**)**. This was found to be driven by a significant increase in the number and length of secondary neurites **(**Supplemental Material, **Fig S7D****)**. Given the immaturity of these differentiating neurons, these neurites are likely nascent dendrites emerging prior to axo-dendritic specification rather than axons or dendrites specifically. Collectively, these data demonstrate NLGN3 and NLGN4X can drive morphological changes in hNPCs and significantly promote neurite outgrowth in immature human neurons, consistent with their subcellular distribution. Critically, ASD mutants of these proteins do not exert these effects, which further mirrors their altered subcellular distribution.

### NLGN3 affects growth cone structure by influencing actin filament alignment

**Figure 5:**
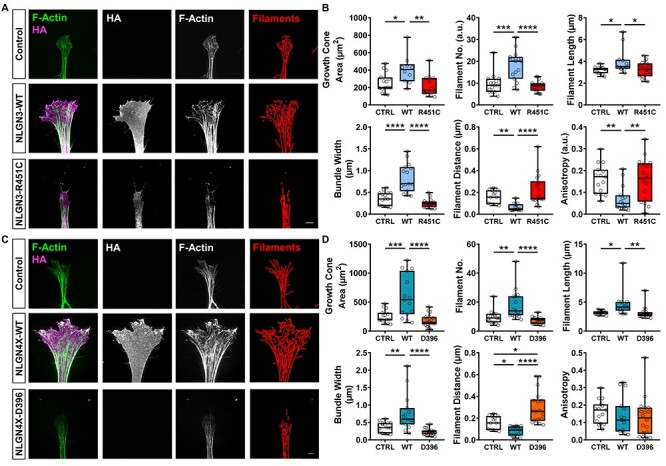
NLGN3/4X-WT effects growth cone structure by influencing actin filament organization. (A) Representative super-resolution images showing ectopic wildtype (WT) NLGN3 expression influences growth cone area and actin filament organization. Scale bar = 5 μm. (B) Data showing ectopic NLGN3-WT expression significantly increases growth cone area, filament number, filament length and filament bundle width while decreasing filament distance and anisotropy compared to control or ectopic NLGN3-R451C expression, confirmed by parametric one-way ANOVA with Bonferroni post-hoc correction or non-parametric Kruskal–Wallis *H* test with Dunn’s post-hoc test (*n* = 15). (C) Representative super-resolution images showing ectopic NLGN4X-WT expression influences growth cone area and actin filament organization. Scale bar = 5 μm. (D) Data showing ectopic NLGN4X-WT expression significantly increases growth cone area, filament number, filament length and filament bundle width while decreasing filament distance but not anisotropy compared to control or ectopic NLGN4X-D396 expression, confirmed by parametric one-way ANOVA with Bonferroni post-hoc correction or non-parametric Kruskal–Wallis *H* test with Dunn’s post-hoc test (*n* = 15). Filament distance was also found to be significantly increased between control and ectopic NLGN4X-D396 conditions suggesting a dominant negative effect.

The primary driver and navigator of neuritogenesis is the growth cone (4). The growth cone is a highly dynamic actin-rich structure. Therefore, we hypothesized that ectopic NLGN3-WT or NLGN3-R451C would influence growth cone morphology and actin filaments. Investigation of growth cones by super-resolution imaging revealed that ectopic NLGN3-WT but not NLGN3-R451C expression significantly increased growth cone area **(**Fig 5A and B**)**. This relative lack of growth cone expansion in the NLGN3-R451C condition may be a consequence of the decrease in ectopic NLGN3 clusters at the growth cone leading edge, also indicating the mutant does not interfere with endogenous NLGNs. As growth cone area was found to be significantly correlated to the number of actin filaments within the growth cone **(**Supplemental Material, **Fig S8A****)**, we then looked at whether NLGN3 exerted any influence on actin filaments. Indeed, ectopic NLGN3-WT but not NLGN3-R451C significantly increased growth cone filament number and significantly decreased the distance between growth cone filaments **(**Fig 5B**)**. Additionally, a significant decrease in anisotropy of actin filaments was found between the NLGN3-WT condition and control; i.e. actin filaments in the growth cones of immature neurons ectopically expressing NLGN3-WT are significantly more parallel than actin filaments in immature neurons in control or NLGN3-R451C conditions **(**Fig 5A and B**)**. Combined, these data suggest NLGN3 has a profound effect on both broad and specific growth cone morphology as well as modulating actin filament orientation within the growth cone.

### NLGN4X affects growth cone structure by influencing actin filament organization

We also noticed NLGN4X-WT influencing growth cone morphology and actin filaments in similar ways as NLGN3-WT. We reasoned that these functional similarities are as a consequence of the relatively high protein sequence homology shared between NLGN3 and NLGN4X. Similar to ectopic NLGN3-WT expression, ectopic NLGN4X-WT expression increased growth cone area compared to control **(**Fig 5C and D**)**. No such growth cone expansion was observed in the NLGN4X-D396 condition. This relative lack of growth cone expansion in the NLGN4X-D396 condition may be as a consequence of the almost total lack of ectopic NLGN4X clusters at the growth cone leading edge, also indicating the mutant form does not interfere with endogenous NLGNs. As growth cone area was found to be significantly correlated to the number of actin filaments within the growth cone **(**Supplemental Material, **Fig S8B****)**, we then looked at whether NLGN4X exerted any influence on actin filaments, similar to NLGN3. Ectopic NLGN4X-WT expression was found to significantly increase growth cone filament number and significantly decreased the distance between growth cone filaments when normalized to filament number **(**Fig 5D**)**. Additionally, these data revealed a dominant negative effect for the NLGN4X-D396 mutation as a significant increase in filament distance was found in the NLGN4X-D396 condition compared to control **(**Fig 5D**);** i.e. NLGN4X-D396 negatively influences the distance between actin filaments within growth cones. However, contrary to the NLGN3-WT anisotropy data, no significant differences in anisotropy were detected between any NLGN4X condition **(**Fig 5D**)**. Taken together, these data suggest NLGN3 and NLGN4X have profound effects on growth cone F-actin remodeling, ultimately leading to significant changes in growth cone size and structure. These data also infer a potential link between NLGN3/4X nanodomain clustering, growth cone F-actin, and actin regulator proteins.

### NLGN3/4X regulates PAK1 phosphorylation via an interaction with Shank3

**Figure 6:**
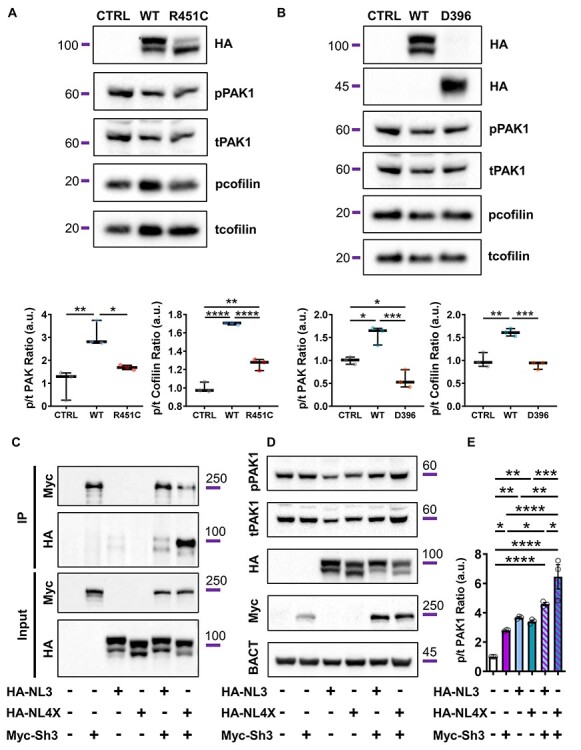
NLGN3/4X-WT induces p21-activated kinase (PAK1) phosphorylation, likely via Shank3. (A) Representative blots and data showing ectopic NLGN3-WT expression in HEK293 cells increases phosphorylation of actin regulator proteins PAK1 and cofilin, confirmed by parametric one-way ANOVA with Bonferroni post-hoc correction (*n* = 3). (B) Representative blots and data showing ectopic NLGN4X-WT expression in HEK293 cells increases phosphorylation of actin regulator proteins PAK1 and cofilin, confirmed by parametric one-way ANOVA with Bonferroni post-hoc correction (*n* = 3). (C) Co-immunoprecipitation data showing NLGN3 and NLGN4X interact with Shank3 in HEK293 cells co-transfected with HA-NLGN3/4X-WT and Myc-Shank3-WT. (D + E) Representative blots and data showing ectopic co-expression of HA-NLGN3/4X-WT and Myc-Shank3-WT has a compounding effect on PAK1 phosphorylation compared to untransfected control and single transfection conditions, confirmed by parametric one-way ANOVA with Bonferroni post-hoc correction (*n* = 3).

Previous studies have shown that PAK1 modulates neurite outgrowth, polarity and establishing overall neuronal morphology through the regulation of downstream actin regulators within the growth cone (2,6). We therefore hypothesized that the signalling pathway underlying NLGN3 and NLGN4X’s ability to regulate neurite outgrowth and growth cone dynamics may be mediated by PAK1. To test this hypothesis, we ectopically expressed NLGN3/4X constructs in HEK293 cells and assessed phospho-PAK1 (pPAK1) and phospho-cofilin (pcofilin) levels, which is indicative of their ability to remodel actin and can therefore be used as proxies for actin dynamics due to their involvement in actin treadmilling (6,35). This revealed significant increases in PAK1 and cofilin phosphorylation between control and NLGN3-WT (~100 kDa) conditions but no significant difference in phosphorylation for either PAK1/cofilin in the NLGN3-R451C (~100 kDa—SNP) condition **(**Fig 6A**)**. Significant increases in PAK1 and cofilin phosphorylation were also found between control and NLGN4X-WT (~100 kDa) conditions while no significant differences in phosphorylation were found for the NLGN4X-D396 (~45 kDa—truncation) condition **(**Fig 6B**)**. These changes in PAK1 phosphorylation suggest that NLGN3/4X regulate PAK1 phosphorylation, which may underlie their ability to regulate the cytoskeleton.

We next further investigated the mechanism by which NLGN3/4X regulated PAK1 phosphorylation. Previous studies have demonstrated that NLGNs interact with Shank3 via their C-terminals (36,37). PAK1 has also been shown to interact with Shank3 via a βPIX/Rac1/Cdc42-dependent pathway (38,39). Shank3 haploinsufficiency influences neurite outgrowth in human neurons (20) and has previously been shown to localize to growth cones (40,41). We therefore hypothesized an interaction between NLGN3, NLGN4X and Shank3 linked NLGN3/4X to PAK1. Consistent with previous reports, co-immunoprecipitation studies confirmed that Myc-Shank3 and HA-NLGN3-WT or NLGN4X-WT formed protein complexes **(**Fig 6C**)**. To demonstrate a role of this protein complex in regulating PAK1 phosphorylation, we ectopically co-expressed HA-NLGN3/4X-WT and Myc-Shank3 constructs in HEK293 cells and assessed pPAK1 levels. This revealed significant increases in PAK1 phosphorylation between control and all single expression conditions. However, a compounding effect of Shank3 and NLGN3/4X co-expression on PAK1 phosphorylation was also observed **(**Fig 6D**and E)**. This compounding effect was found to be significantly higher in the Shank3/NLGN4X co-expression condition than the Shank3/NLGN3 co-expression condition, further indicating a potential functional divergence between NLGN3 and NLGN4X in their signalling mechanisms. These changes in PAK1 phosphorylation suggest that NLGN3/4X regulate PAK1 phosphorylation potentially via an interaction with Shank3.

### Effects of NLGN3/4X on growth cone structure are attenuated by PAK1 inhibition

**Figure 7:**
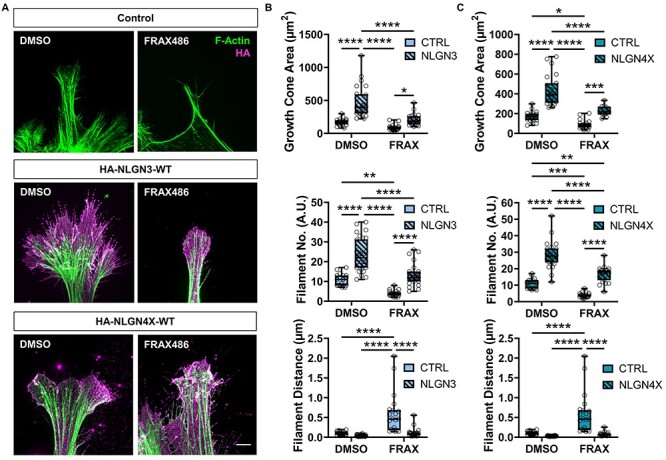
Growth cone morphology changes induced by NLGN3/4X-WT are attenuated by PAK1 inhibition. (A) Representative images showing increases in growth cone area, filament number and decreases in filament distance induced by ectopic NLGN3/4X-WT expression are attenuated by PAK1 inhibition. Scale bar = 5 μm. (B) Data showing increases in growth cone area, filament number and decreases in filament distance induced by ectopic NLGN3-WT expression are attenuated by PAK1 inhibition, confirmed by two-way ANOVA with Bonferroni post-hoc correction (*n* = 25). (C) Data showing increases in growth cone area, filament number and decreases in filament distance induced by ectopic NLGN4X-WT expression are attenuated by PAK1 inhibition, confirmed by two-way ANOVA with Bonferroni post-hoc correction (*n* = 25).

To demonstrate NLGN3/4X-induced PAK1 phosphorylation was required for changes in growth cone size, we ectopically expressed NLGN3-WT or NLGN4X-WT and cotreated with a PAK1 activity inhibitor, FRAX486 (42,43). Treating immature neurons with 50 nM FRAX486 in the absence of NLGN3/4X significantly decreased growth cone area by 49% in control cells compared to vehicle (DMSO) treated cells, as anticipated given the crucial role of PAK1 in controlling growth cone morphology **(**Fig 7A**)**. Conversely, immature neurons ectopically expressing NLGN3-WT cotreated with FRAX486, showed a non-significant increase in growth cone size compared to control DMSO treated cells **(**Fig 7A and B**)**. A similar pattern was observed for filament number i.e. increases in filament number induced by ectopic NLGN3-WT expression are attenuated by FRAX486 cotreatment. Lastly, the decrease in filament distance induced by NLGN3-WT ectopic expression was also attenuated by FRAX486 cotreatment **(**Fig 7A and B**)**. Taken together, these data implicate PAK1 pathway activation as a core component of the molecular mechanism underlying the previously observed subcellular phenotypes induced by ectopic NLGN3-WT expression and demonstrate a causal association between NLGN3, PAK1, and growth cone actin organization.

Inhibiting PAK1 had a similar effect on NLGN4X-induced remodeling of growth cones and actin. Indeed, cotreatment with FRAX486 (50 nM) attenuated NLGN4X-WT-induced enlargement of growth cone area **(**Fig 7A and C**)**. Cotreatment with FRAX486 also partially blocked increases in filament number induced by ectopic NLGN4X-WT expression. Lastly, the decrease in filament distance induced by ectopic NLGN4X-WT expression was attenuated by FRAX486 cotreatment **(**Fig 7A and C**)**. Combined, these data implicate PAK1 pathway activation as a core component of the molecular mechanism underlying the previously observed subcellular phenotypes induced by ectopic NLGN4X-WT expression and further demonstrate a causal link between NLGN4X, PAK1, and growth cone actin organization.

### PAK1 inhibition decreases NLGN3/4X growth cone clustering

**Figure 8:**
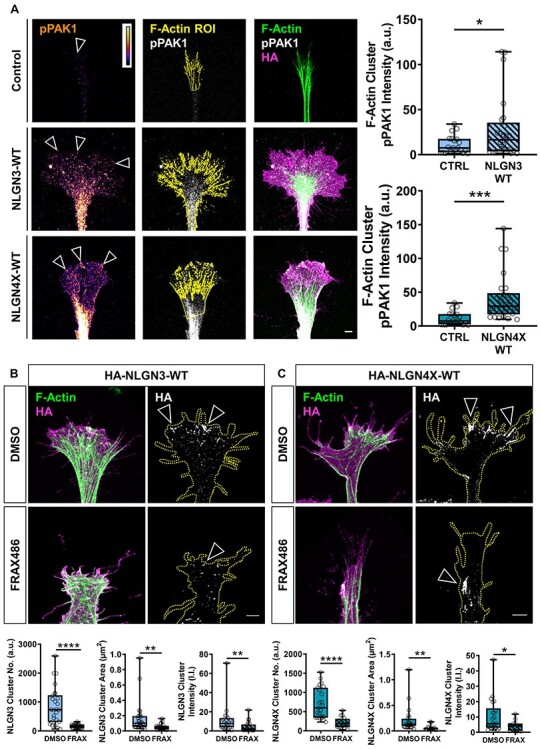
NLGN3/4X-WT clustering at growth cones is dependent on PAK1 signalling. (A) Representative images and data showing phosphorylated PAK1 intensity significantly increases in growth cone F-actin clusters of immature neurons ectopically expressing NLGN3/4X-WT, confirmed by *t*-test. Scale bar = 5 μm. (B) Representative images and data showing ectopically expressed HA-NLGN3-WT clusters are attenuated by PAK1 inhibition for cluster number, area, and intensity, confirmed by *t*-test (*n* = 25). Scale bar = 5 μm. (C) Representative images and data showing ectopically expressed HA-NLGN4X-WT clusters are attenuated by PAK1 inhibition for cluster number, area, and intensity, confirmed by *t*-test (*n* = 25). Scale bar = 5 μm.

To validate that NLGN3/4X increased pPAK1 in immature neurons we ectopically expressed NLGN3/4X-WT in immature neurons and measured pPAK1 levels specifically at F-actin/ NLGN3/4X clusters. Phospho-PAK1 intensity significantly increased in growth cone F-actin clusters of immature neurons ectopically expressing NLGN3-WT or NLGN4X-WT **(**Fig 8A**)**. These data are in line with the immunoblotting data and provide evidence that NLGN3 and NLGN4X clustering strongly induces PAK1 phosphorylation.

We previously observed that NLGN3/4X-WT formed nanoscopic clusters at the growth cone leading edge where it colocalized with actin. Conversely, mutant forms of these proteins displayed reduced localization in growth cones and impaired ability to remodel growth cone morphology and actin organization. Interestingly, inhibiting PAK1 signalling with FRAX486 produced growth cone morphologies and actin organization similar to that produced by mutant NLGN3/4X. Therefore, we hypothesized that treatment with FRAX486 may also disrupt the nanoscopic clustering of ectopic NLGN3/4X-WT within growth cones. To examine this, we compared the abundance of either adhesion protein at the leading edge of growth cones with or without FRAX486 cotreatment. This revealed significant decreases in NLGN3-WT growth cone cluster number, area and intensity in FRAX486 compared to DMSO treated cells **(**Fig 8B**)**. This decrease remained significant even when cluster number was normalized to growth cone area **(**Supplemental Material, **Fig S9A****)**. A similarly significant decrease was also observed for NLGN4X-WT growth cone clusters for number, area and intensity when treated with FRAX486 compared to DMSO **(**Fig 8C**)**. This decrease also remained significant when cluster number was normalized to growth cone area **(**Supplemental Material, **Fig S9B****)**. Combined, these data suggest PAK1 activation also plays a role in clustering NLGN3/4X at the growth cone promoting its adhesive function. Furthermore, combined with previous data, this not only suggests NLGN3/4X are capable of activating PAK1 in the growth cone but also PAK1 activation can promote NLGN3/4X clustering in growth cones.

### Effects of NLGN3/4X on neurite outgrowth are attenuated by PAK1 inhibition

**Figure 9:**
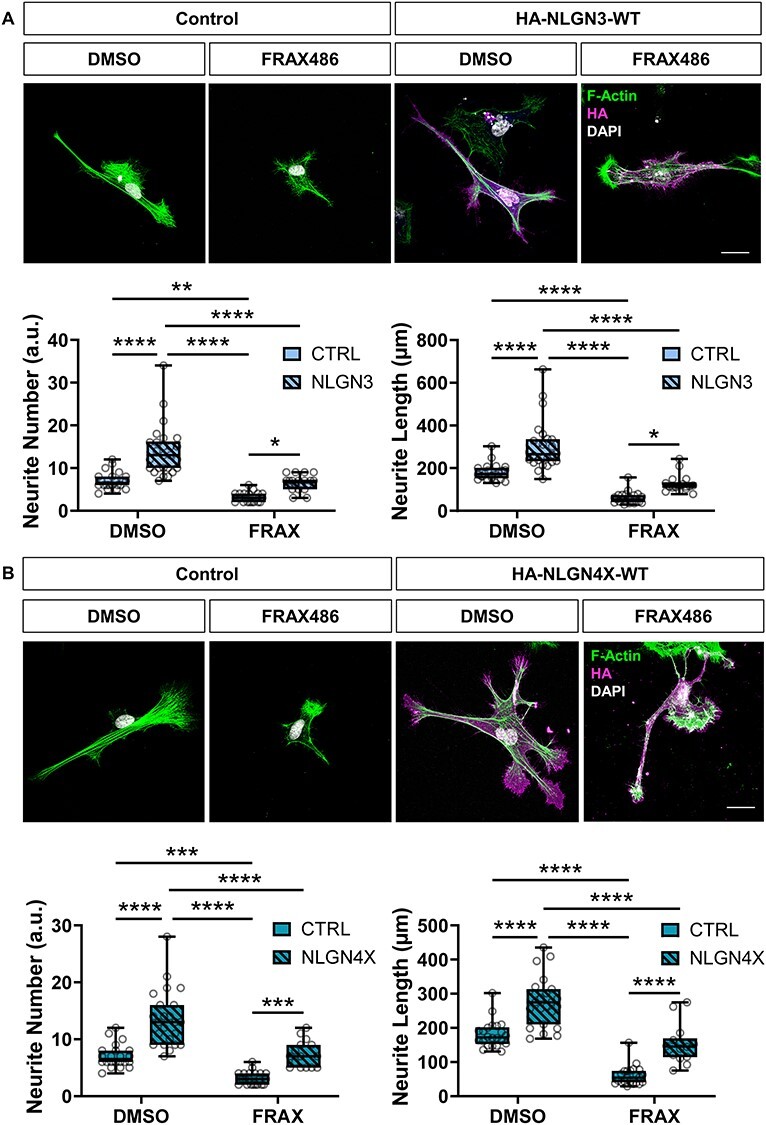
NLGN3/4X-WT dependent neuritogenesis is dependent on PAK1 phosphorylation. (A) Representative images and data showing ectopic NLGN3-WT mediated increases in neurite number and length are attenuated by PAK1 inhibition, confirmed by two-way ANOVA with Bonferroni post-hoc correction (*n* = 25). Scale bar = 25 μm. (B) Representative images and data showing ectopic NLGN4X-WT mediated increases in neurite number and length are attenuated by PAK1 inhibition, confirmed by two-way ANOVA with Bonferroni post-hoc correction (*n* = 25). Scale bar = 25 μm.

Given that FRAX486 attenuated the nanoscopic clustering of ectopic NLGN3/4X-WT in growth cones, we hypothesized that this would also impact the overall morphology of the cell. Consistent with this notion, FRAX486 treatment significantly decreased neurite number and length in immature neurons compared to DMSO controls. FRAX486 cotreatment inhibited NLGN3-WT-induced effects on neurite number and length **(**Fig 9A**)**. In agreement with previous data, ectopic NLGN4X-WT expression resulted in significantly increased neurite number and length. However, again, cotreatment with FRAX486 blocked NLGN4X-WT’s effect on neuritogenesis, attenuating neurite number and length **(**Fig 9B**)**. Taken together, these data indicate that clustering of NLGN3/4X at the leading edge of growth cones is required for both increased growth cone size and neuritogenesis via a PAK1-dependent mechanism.

## Discussion

**Figure 10:**
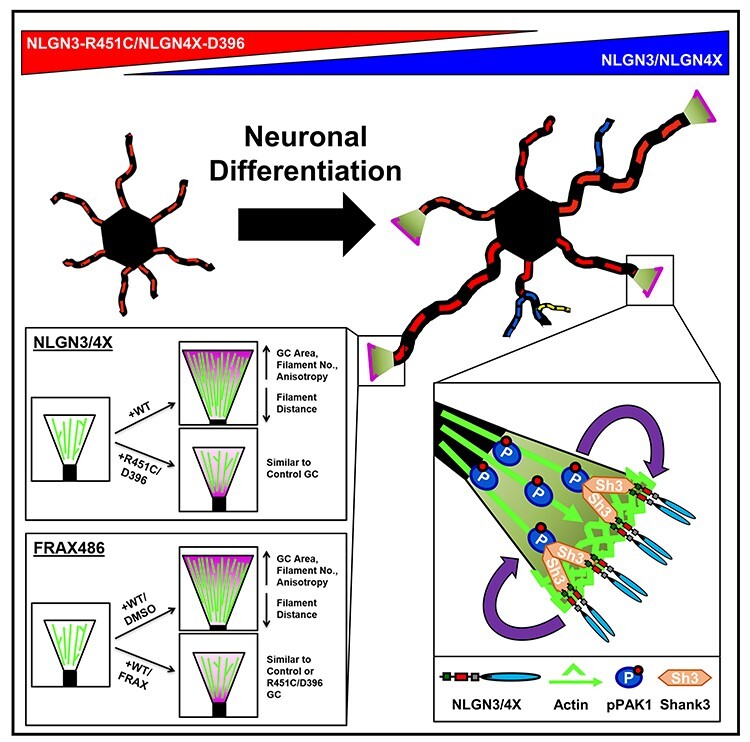
Model of NLGN3/4X clustering on growth cones and neurites dependent on NLGN-clustering/PAK1 feedback loop. Schematic diagram summarizing all primary findings of this research including the effects on neuritogenesis (upper—primary neurites = red, secondary neurites = blue, tertiary neurites = yellow), the effects on growth cone actin (lower left), and the molecular mechanism involved in both phenotypes (lower right). NLGN, neuroligin; GC, growth cone; WT, wild type; DMSO, dimethyl sulfoxide; FRAX, FRAX486; PAK1, p21-activated kinase.

CAMs are key regulators of neuritogenesis, particularly at the growth cone leading edge (44). Gene mutations in the trans-synaptic neurexin-neuroligin cell-adhesion complex are frequently associated with individuals with ASD, particularly neuroligin-3 and neuroligin-4X (NLGN3/4X) (13). However, the role of NLGN3/4X during human cellular neurodevelopment is largely unknown. Furthermore, the functional consequences of ASD-associated NLGN3/4X mutations have yet to be investigated in human neurodevelopment. Here we demonstrate novel roles for NLGN3/4X in early human neurodevelopment. Specifically, we demonstrate NLGN3 and NLGN4X nanodomains promote neuritogenesis during cellular neurodevelopment via actin filament organization within the growth cone, mediated by PAK1 signalling. We also observed that growth cones of immature neurons ectopically expressing NLGN3/4X-WT cotreated with the PAK1 phosphorylation inhibitor, FRAX486, appeared similar in size and structure to growth cones ectopically expressing NLGN3-R451C or NLGN4X-D396. Furthermore, we demonstrate a novel feedback loop between PAK1 and NLGN3/4X nanodomain clustering which contributes to this growth cone enlargement and, by extension, neuritogenesis. Our data reveal novel roles for both NLGN3 and NLGN4X in the development of human cortical neurons, which is not replicated by ASD-associated mutants of these adhesion proteins **(**Fig 10**)**.

The similarity in effect and signalling pathways activated by NLGN3/4X is consistent with the comparatively high amino acid sequence homology between NLGN3 and NLGN4X (69.026% homology) (45,46) and the finding that NLGN4 evolved rapidly from other NLGN gene sequences in mice (47). Additionally, NLGN4X overexpression was recently found to rescue aberrant neurite outgrowth induced by ZNF804A knockdown (48). Combined, these suggest necessary roles for both proteins in mammalian neuritogenesis as well as a degree of functional overlap between NLGN3 and NLGN4X. It should also be noted that dendritic outgrowth was found to be increased in Stratum Radiatum neurons in acute hippocampal slices from a NLGN3-R451C knock-in mice (49). However, this increase was not observed in Stratum Oriens or Stratum Lacunosum Moleculare neurons, suggesting this may be a cell type specific effect or could be a compensatory response from other NLGNs. Furthermore, no gross brain structure abnormalities were found in NLGN3-R451C knock-in mice (50). This supports the notion of a cell type specific effect or could suggest a difference between mouse knock-in models and humans with the NLGN3-R451C mutation. This potential species difference would be problematic to examine as human patients with the R451C mutation are rare and NLGN3-R451C human autism patient stem cell lines have yet to emerge. To reconcile this, we also examined neurite outgrowth in dissociated mouse primary cortical neurons and found similar increases in both axon and dendrite number and length when NLGN3/4X-WT were ectopically expressed and, critically, showed a subtle increase in axon length when NLGN3-R451C was ectopically expressed. This could be a consequence of the fact that DIV4 mouse neurons have already established axo-dendritic polarity, whereas DD4 CTX0E16 cells have not (25,51). Furthermore, it is unclear precisely how much of the ectopically expressed NLGN3/4X-WT actually reaches the growth cone cell surface to contribute to adhesion compared to their mutant variants. Future experiments would aim to further investigate these differences.

It is unlikely that NLGN3/4X operate alone in this phenotype. One of the more noteworthy PAK1 binding partners is the ASD-associated protein Shank3 (38,39), which we show here to also interact with both NLGN3 and NLGN4X producing a compounding effect on PAK1 phosphorylation. This finding may also suggest a potential compounding effect of ASD-associated NLGN and SHANK3 mutations influencing neurite outgrowth via PAK1 during neurodevelopment. Alternatively, accumulating evidence has linked NLGNs with other proteins involved in dendrite morphogenesis, such as Rap. Rap proteins are a subfamily of the Ras superfamily of small GTPases. Small GTPases have been extensively implicated in neuritogenesis and as signalling mediators for CAMs (1,2). NLGN3 has previously been shown to interact with the Rap guanine-nucleotide exchange factor, Epac2 (RapGEF4) thus controlling Rap signalling (52). Rare variants in Epac2 have been associated with ASD and have been implicated in impaired basal dendrite morphogenesis (53). It is also noteworthy that NLGN1 can regulate Rap activity via the activation of spine-associated Rap GTPase (SPAR) resulting in the modulation of LIMK/cofilin-mediated actin organization (54). The importance for the correct regulation of such a pathway is highlighted by increasing evidence that ASD-associated mutations in SynGAP, a GAP for Rap, play a significant role in the development of neuronal and synaptic morphology (55). Combined, these findings suggest several ASD-associated proteins operate via different signalling pathways prior to PAK1 but result in a similar modulation of actin organization at the endpoint. Furthermore, this suggests the ability of NLGN3/4X to regulate the actin-cytoskeleton via PAK1 may be mediated by specific binding partners.

The NLGN-PAK1 feedback loop found to be dependent on molecular clustering at the cell membrane was an intriguing discovery and not without precedence. Indeed, filamin, an actin cross-linking protein, was found to operate in a similar bidirectional loop with PAK1 to locally influence actin cytoskeletal dynamics and membrane ruffles (56,57). Furthermore, nanoscale clustering of the chemokine receptor CXCR4 at the leading edge of Jurkat T lymphocyte cells was found to promote dynamic actin rearrangement resulting in increased cell migration (58). Evidence from mature neurons also suggests orchestrated nanoscale recruitment and clustering of CAM at synapses is critical to synaptic function (33). For example, NLGN1 and N-cadherin were found to cooperate to form clusters at synapses in mature hippocampal neurons, ultimately promoting synapse formation (59). This synaptic clustering function was also demonstrated for NLGN3. Mature hippocampal neurons treated with purified Wnt3a exhibited increased NLGN3 recruitment to dendritic processes and, ultimately, increased clustering of NLGN3 with PSD-95 at synapses (31). Lastly, but perhaps most relevantly, transient clustering interactions between flowing actin filaments and immobilized N-cadherin/catenin complexes were demonstrated in neuronal growth cones (7). This led to a local reduction of actin retrograde flow and increased growth cone migration. Combined, this evidence suggests the actin-cytoskeleton responds dynamically to nanoscale clustering of specific molecules at the cell membrane. This response is likely to be similar in growth cones given the high dynamicity of growth cone actin and the integral role of CAMs in the growth cone, therefore, this system may also be a component of neuritogenesis during cellular neurodevelopment. Perturbation of this nanoscale clustering via ASD-associated NLGN mutations may therefore lead to alterations in growth cone motility, ultimately contributing to subtly atypical neurodevelopment. Additionally, the similarity in structure of NLGN3/4X-WT and FRAX486 cotreated growth cones to NLGN3/4X mutant growth cones suggests a link between the molecular mechanism discovered herein and the underlying mechanism that may be involved in the impaired cellular phenotypes seen when the ASD-associated mutant variants are ectopically expressed. Lastly, questions remain regarding extracellular NLGN3/4X binding partners responsible for the phenotypes discovered herein. NLGNs and NRXNs were recently found to be cleaved at the cell surface, the molecular implications of which are being elucidated (60,61). It is possible that a secreted NLGN3/4X or NRXN fragment may be binding to ectopically expressed NLGN3/4X at growth cones to activate actin signalling pathways prior to axo-dendritic specification.

In summary, we demonstrate a novel function for NLGN3/4X during early human neurodevelopment in neuronal growth cones, particularly at the growth cone leading edge. The functional impact of this role at the growth cone was to significantly promote neuritogenesis in immature human neurons. We also illustrate the consequences of this new role on the actin-cytoskeleton in that NLGN3/4X clustering has profound effects on growth cone F-actin remodeling, ultimately leading to significant changes in growth cone size and structure. These functional roles were found to be impaired by ASD-associated mutant forms of NLGN3/4X for which the clustering function was severely impaired. Furthermore, we show a link between NLGN3/4X clustering and PAK1 activation in the growth cone. Ultimately, leading to the discovery of a mechanistic feedback loop between NLGN3/4X and PAK1 in growth cones which drives actin organization, growth cone enlargement, and neuritogenesis.

## Materials and Methods

### Antibodies and plasmids

Antibodies: mouse anti-HA monoclonal (BioLegend, 901 503, 1:1000), rabbit anti-RFP polyclonal (MBL, PM005, 1:500), chicken anti-GFP polyclonal (Abcam, ab13970, 1:1000), chicken anti-Tuj1 polyclonal (Abcam, ab41489, 1:500), rabbit anti-MAP2 (Abcam, ab32454, 1:1000), rabbit anti-Myc (BioLegend, 626 802, 1:500) ActinGreen 488 ReadyProbe (Thermo Fisher, R37110, per manufacturer’s instructions) and rabbit anti-phospho-PAK1 (Ser144)/PAK2 (Ser141) (Cell Signaling Technology, 2606, 1:100). Alexa Fluor 488, 568, 633 (Life Technologies, 1:500) and 4′,6-diamidino-2-phenylindole (DAPI—D1306, Life Technologies, 1:50000) fluorescent secondary antibodies were used in all immunocytochemistry experiments where applicable. Immunoblotting: mouse anti-NLGN3 monoclonal (StressMarq, SMC-471D, 1:1000), rabbit anti-NLGN3 polyclonal (Synaptic Systems, 129 113, 1:1000), rabbit anti-HA polyclonal (ProteinTech, 51 064-2-AP, 1:1000), rabbit anti-NLGN4X monoclonal (Abcam, ab181251, 1:1000), mouse anti-Myc monoclonal (BioLegend, 626 801, 1:200), mouse anti-total cofilin monoclonal (ProteinTech, 66 057-1-lg, 1:3000), rabbit anti-phospho-cofilin (Ser3) polyclonal (Cell Signaling Technology, 3311, 1:1000), rabbit anti-total PAK1/2/3 (Cell Signaling Technology, 2604), rabbit anti-phospho-PAK1 (Ser144)/PAK2 (Ser141) (Cell Signaling Technology, 2606, 1:1000), rabbit anti-HRP (Life Technologies, G-21234, 1:10000), mouse anti-HRP (Life Technologies, A16078, 1:10000) **(**Supplemental Material, **Table S1****)**.

Cloned HA-tagged NLGN3-WT, NLGN3-R451C, NLGN4X-WT and NLGN4X-D396 plasmids were gifts from Prof. Peter Scheiffele (23). A pmCherry-N1 plasmid (ClonTech, 632 523) was utilized as a morphological marker in HEK293 immunocytochemistry experiments. A peGFP-N2 plasmid (ClonTech, 632 483) was utilized as a morphological marker in subsequent proliferating CTX0E16 experiments due to red fluorescent protein aggregation in CTX0E16 human neural progenitor cells.

### Cell culture and transfection

Mouse cortical neuronal cultures were prepared from CD1 mice E15 embryos. Experimental procedures were carried out in accordance with the Home Office Animals (Scientific procedures) Act, UK, 1986. All animal experiments were given ethical approval by the ethics committee of King’s College London (UK). Briefly, embryos were removed, cortices were dissected, then dissociated in 0.25% trypsin (Gibco 25 200). Cells were plated onto 18 mm glass coverslips (0117580, Marienfeld-Superior GmbH & Co.), coated with poly-d-lysine (0.2 mg/ml, Sigma), at a density of 3 × 105/well equal to 857/mm^2^ and cultured in B27 supplemented neurobasal medium. Neuronal culture media composition can be found in the Supplementary Information. Freshly plated neurons were transfected with GFP and HA-NLGN3/4X constructs on DIV (days in vitro) 1 via lipofectamine, fixed and stained on DIV4, and imaged using epifluorescent microscopy. Neurites were quantified via NeuronJ.

The CTX0E16 human neural progenitor cell line (hNPCs) was obtained from ReNeuron Ltd (Guildford, UK) under a Material Transfer Agreement between ReNeuron and King’s College London. CTX0E16 hNPCs were maintained in 4-hydroxytamoxifen (Sigma, H7904) supplemented DMEM:F12 medium (Sigma, D6421) and were neuralized in Neurobasal medium (Invitrogen, 12 348 017) supplemented with serum-free B27 (Life Technologies, 17 504 044) without 4-hydroxytamoxifen (51,62). HNPCs were plated on PDL/laminin coated 1.5H glass coverslips in neuralization media at 12 500 cells per well and transfected 24 hours later with 2 μg of each human HA-NLGN construct using Lipofectamine 2000 (Invitrogen, 17 504 044). Immature neurons were fixed 4 days post-neuralization and stained using immunocytochemistry.

### Super-resolution microscopy

Transfected immature neurons grown on 1.5H glass coverslips were fixed in 4% formaldehyde/2% sucrose in PBS and subjected to immunocytochemistry. Super-resolved structured illumination microscopy (SIM) images of growth cones were collected using an instant (i)SIM (Nikon) equipped with a 100× TIRF 100× lens (NA 1.49). Prior to any super-resolution image acquisition, the TIRF lens was calibrated using TetraSpeck 0.1 μm Microspheres (Invitrogen, T7279) per manufacturer’s instructions to ensure sub-micron accuracy during acquisition **(**Supplemental Material, Fig S8**)**. Point spread function data were calculated using the ImageJ plug-in PSFj (Theer et al., 2014). Once calibrated, multiple images per stack were collected spaced apart by 0.05 μm. Data were deconvolved using a Richardson-Lucy algorithm specific to the iSIM mode of imaging to increase contrast and resolution using the supplied NIS-Elements Advanced Research software.

### Immunoblotting

Cultured cells were lysed in lysis buffer consisting of 20 mM Tris; pH 7.2, 150 mM NaCl, 1% Triton-X-100, 5 mM EDTA; pH 8, 0.1% SDS, 1% sodium deoxycholate with additional phosphatase inhibitors (Sigma, P0044). Detergent soluble lysates were sonicated and centrifuged to remove cell debris. Samples were resolved by SDS-PAGE, transferred to a nitrocellulose or PVDF membrane and blocked for 1 hour in 5% bovine serum albumin (Sigma, A7906) in TBS-T. Membranes were then immunoblotted with primary antibodies overnight at 4°C, followed by incubation with anti-mouse or anti-rabbit horseradish peroxidase (HRP) conjugated secondary antibodies for 1 h at room temperature. Membranes were then incubated in Clarity electrochemiluminescence substrate (Bio-Rad, 1 705 061) for 5 min and subsequently scanned using the Bio-Rad ChemiDoc MP (Bio-Rad). Band intensity was quantified by densitometry using Image Lab software (Bio-Rad, v6.0).

### RNA isolation, cDNA synthesis, RT-PCR and qPCR

Proliferating hNPCs (*n* = 3) or differentiating immature neurons (*n* = 3) CTX0E16s were pelleted and lysed in TRI Reagent (Ambion, AM9738). Total RNA was then extracted per the manufacturer’s instructions. Residual genomic DNA was removed from each of six biological replicates using the TURBO DNA-*free* Kit (Life Technologies, AM1907) per the manufacturer’s instructions. cDNA was synthesized from 1 μg of total RNA from each extraction using random decamers (Ambion, AM5722G) and SuperScript III Reverse Transcriptase (Invitrogen, 18 080 044), per the manufacturer’s instructions.

To determine the expression of specific genes, primers were designed to target all known RefSeq transcripts of genes of interest, sourced from the UCSC Genome Browser website (http://genome.ucsc.edu) **(**Supplemental Material, **Table S2****)**. Primers were designed to span intronic regions of the selected genes to ensure specific amplification of mRNA, even in the presence of DNA contamination. Reactions were carried out in a total volume of 20 μl containing diluted cDNA, 1 X HOT FIREPol Blend Master Mix (Solis Biodyne, 04-25-00125) and primers at 200 nM, using a GS4 thermal cycler. Samples were separated and visualized by agarose gel electrophoresis.

For quantitative expression analysis, 20 μl cDNA samples from SuperScript III reactions were diluted with a further 120 μl of nuclease-free H_2_O. Reactions were carried out in a total volume of 20 μl, containing diluted cDNA, 1× HOT FIREPol EvaGreen q-PCR Mix (Solis Biodyne, 08-25-00001) and primers at 200 nM, using an MJ Research Chromo 4 (Bio-Rad) and MJ Opticon Monitor analytic software (Bio-Rad). Triplicate qPCR reactions were performed to measure each gene in each cDNA sample. The level of each gene was measured against a standard curve constructed by serial dilution of pooled cDNA from all assayed samples. A relative value was thus obtained for each of the three triplicate reactions for each cDNA sample. Mean measures of target genes were then normalized against a geometric mean determined from 2 internal control genes (ALG2 & RPL6) for each cDNA sample to yield a relative target gene expression value for all samples. ALG2 & RPL6 were identified as suitable internal controls based on a combination of previous whole-genome microarray data of CTX0E16 cells, where it showed the least variability (in terms of standard deviation) across conditions and a housekeeper screen qPCR. Normalized qPCR target gene expression values were compared between hNPC or immature neuron CTX0E16 cells.

### Quantification

All neurite outgrowth images were quantified using the NeuronJ ImageJ plug-in (http://www.imagescience.org/meijering/software/neuronj/ v1.4.2) which allowed for manual tracing and labelling (primary, secondary or tertiary) of individual neurites (63) **(**Supplemental Material, **Fig S8C****)**. Unless otherwise stated, all experiments were repeated for at least three biological replicates, complete information on all statistical analysis and experimental replication can be found in Supplementary Information—Quantification and Statistical Analysis.

Growth cone area was quantified using NIS-Elements Advanced Research software. Growth cone filaments were quantified using line scan analysis in ImageJ **(**Supplemental Material, **Fig S8D and E)**. Growth cone filament skeletons were generated using the ImageJ plug-in Ridge Detection (64). Growth cone filament anisotropy was quantified using the ImageJ plug-in FibrilTool (65). All datasets were subjected to the ROUT method of outlier detection, detected outliers were subsequently removed. All data and error bars are shown as mean ± standard error of the mean to two decimal places. A detailed summary of statistics can be found in Supplemental Material, **Table S3**; details of morphological quantification can be found in Supplemental Material, Tables S4 and S5.

## Supplementary Material

Gatford_etal_SupplementaryInformation_ddab277

## Data Availability

All data supporting the findings of this manuscript are available from the corresponding authors upon reasonable request. NLGN3/4X mRNA expression data are freely available from the BrainSpan Atlas of the Developing Human Brain compiled primarily by the Allen Institute for Brain Science. A non-peer reviewed version of this manuscript has been posted on the preprint server BioRxiv (doi: https://doi.org/10.1101/546499).
