# Supplementary material for: Neuroligin-3 and neuroligin-4X form nanoscopic clusters and regulate growth cone organization and size
Source: Hum Mol Genet. 2021 Sep 20;31(5):674–91. doi: 10.1093/hmg/ddab277 (PMC8895740; doi:10.1093/hmg/ddab277)
Supplement: Gatford_etal_SupplementaryInformation_ddab277 [file gatford_etal_supplementaryinformation_ddab277.zip › Gatford_etal_SupplementaryInformation_ddab277.pdf]

# Neuroligin-3 and Neuroligin-4X Form Nanoscopic Clusters and Regulate Growth Cone Organization and Size

Nicholas J. F. Gatford[1], P. J. Michael Deans[1], Rodrigo R.R. Duarte[1], George Chennell[1], Katherine J. Sellers[1], Pooja Raval[1], Deepak P. Srivastava[1,2]*

***Supplemental Information***

## SUPPLEMENTAL MATERIALS AND METHODS

### *Experimental Design*

The objective of these controlled laboratory experiments was to better understand the role of NLGN3 and NLGN4X during human neurodevelopment using human neural progenitor cells and young human neurons. The original hypothesis was that NLGN3 and NLGN4X promote neuritogenesis in human neurodevelopment. All further hypotheses were generated after this initial hypothesis was proven. Sample size was chosen based on the minimum necessary number of biological replicates to conduct inferential statistical testing. However, data from 5 technical replicates were gathered and averaged per biological replicate to ensure accuracy and reliability. Data were collected in all experiments until this threshold was reached. No sample size calculation was performed, no data were excluded from any dataset, and outliers were not removed. All cells were allocated randomly into groups when plated at the start of each experiment. Cells were plated at the same approximate density between groups based on cell count calculations. Blinding during data collection or analysis was not possible as the phenotypes being investigated were difficult to

hide with conventional blinding methods. All cell lines tested negative for mycoplasma contamination.

For antibody validation, three biological replicate whole cell lysate samples were generated (see immunoblotting section) and separated per lane. One biological replicate was considered as a single passage/plating between flasks of fully confluent cells. For PAK/cofilin mechanism experiments, three biological replicate whole cell lysate samples per condition (control, wildtype NLGN, and mutant NLGN) were generated 48 hours post-transfection. The protrusion outgrowth experiment was conducted in HEK293 cells once to confirm the outgrowth phenotype.

For endogenous NLGN3/4X expression experiments, three biological replicate whole cell protein or RNA lysates per time point (NPC and neuron) were generated in parallel (see immunoblotting or RNA isolation sections). Super-resolution images of endogenous NLGN3/4X expression were conducted once to assess endogenous protein localisation in immature neurons.

Super-resolution assessment of nanodomains was conducted once to confirm their presence in and at the leading edge of growth cones. For neurite outgrowth experiments, five images per condition (control, wildtype NLGN, mutant NLGN) were acquired and quantified for each of three biological replicates. One biological replicate was considered as a single passage/plating between flasks of fully confluent cells. Neurites measuring less than 3 µm were excluded from analysis as these were deemed too small to constitute a neurite. Line scan analyses of exogenously expressed growth cone and neurite NLGN3/4X were conducted once to demonstrate the localisation disparity between wildtype and mutant NLGN. For super-resolution growth cone actin analyses, five images per condition (control, wildtype NLGN, mutant NLGN) were acquired and quantified for each of three biological replicates (see microscopy and quantification sections). For super-resolution

analysis of growth cone pPAK1 and wildtype NLGNs, five images per condition (control, wildtype NLGN) were acquired and quantified for each of three biological replicates.

For super-resolution growth cone FRAX486 attenuation experiments, five images per condition (control DMSO, control FRAX, NLGN DMSO, NLGN FRAX) were acquired and quantified for each of three biological replicates. For FRAX486 treated growth cone NLGN cluster experiments, five images per condition (control DMSO, control FRAX, NLGN DMSO, NLGN FRAX) were acquired and quantified for each of three biological replicates. For neurite FRAX486 attenuation experiments, five images per condition (control DMSO, control FRAX, NLGN DMSO, NLGN FRAX) were acquired and quantified for each of three biological replicates.

***Cell Culture and Transfection***

Antibody validation, protrusion outgrowth, and PAK/cofilin mechanism experiments used human embryonic kidney cells (HEK293) cultured in DMEM:F12 (Sigma, D6421) supplemented with 10% foetal bovine serum (ClonTech, 631107) and 1% L-glutamine (Sigma, G7513), in a 37°C/5% $CO_2$ atmosphere. Cells were seeded at 30-40% confluence on PDL/laminin coated acid-washed 1.5H glass coverslips 24 hours before transfection (1).

The conditionally immortalised cortically derived human neural progenitor cell line (hNPC) CTX0E16 was obtained from ReNeuron Ltd. (Guildford, UK) under a Material Transfer Agreement between ReNeuron and King's College London. CTX0E16 hNPCs were derived from 12-week foetal cortical neuroepithelium and conditionally immortalized using a c-mycER$^{TAM}$ transgene. Characterization of the CTX0E16 cell line is described in detail elsewhere (1, 2). Proliferation and neuralization of CTX0E16 cells were carried out as previously described (1). Briefly, CTX0E16 cells were neuralized by replacing 4-hydroxytamoxifen (Sigma, H7904)

supplemented DMEM:F12 medium (Sigma, D6421) with Neurobasal medium (Invitrogen, 12348017) supplemented with serum-free B27 (Life Technologies, 17504044).

Mouse cortical neuronal cultures were prepared from CD1 mice E15 embryos. Animals were habituated for 3 days before experimental procedures, which were carried out in accordance with the Home Office Animals (Scientific procedures) Act, United Kingdom, 1986. All animal experiments were given ethical approval by the ethics committee of King's College London (United Kingdom). Briefly, the embryos were removed and placed in ice-cold Hanks' balanced salt solution, cortices were dissected and dissociated in 0.25% trypsin (Gibco 25200). Cells were plated onto 18 mm glass coverslips (No 1.5; 0117580, Marienfeld-Superior GmbH & Co.), coated with poly-d-lysine (0.2 mg/ml, Sigma), at a density of 3 × 105/well equal to 857/mm$^2$. Neurons were cultured in feeding media: neurobasal medium; minus phenol red (12349-015) supplemented with 2% B27 (17504044), 1% GlutaMAX Supplement (35050-061) and 1% penicillin:streptomycin (15070063), 1% Sodium pyruvate (11360-070) (all reagents from Life Technologies). Freshly plated neurons were transfected with GFP and HA-NLGN3/4X constructs on DIV (days in vitro) 1 following an identical procedure to the CTX0E16 experiments, fixed and stained on DIV4, and imaged using a Zeiss Axio Imager Z1 epifluorescent microscope. Neurites were measured and labelled using NeuronJ.

All transfections in HEK293, CTX0E16 hNPCs, and mouse primary cortical neurons were carried out using Lipofectamine 2000 (Invitrogen, 17504044), per the manufacturer’s instructions. Briefly, 2 µg of each HA-NLGN and 2 µg of peGFP-N2, mCherry, or Myc-Shank3-WT (where applicable) construct were mixed with 2 µl of Lipofectamine 2000 in 100 µl DMEM:F12 and incubated for 20 minutes in a 37°C/5% $CO_2$ atmosphere. The DNA:Lipofectamine 2000 mixture was added dropwise to HEK293, CTX0E16 hNPCs, or mouse primary cortical neurons which

were then incubated for 4 hours at 37°C, before being transferred to new wells containing fresh media. Proliferating HEK293 or CTX0E16 hNPCs recovered for 48 hours in an incubator at 37°C/5% CO2 prior to fixation and immunocytochemistry. Differentiating CTX0E16 hNPCs and mouse primary cortical neurons recovered for 48 hours and continued differentiating for a further 24 hours in an incubator at 37°C/5% $CO_2$ prior to fixation and immunocytochemistry.

The brain penetrant and orally bioavailable p21-activated kinase (PAK) inhibitor FRAX486 (Tocris, 5190) was used to inhibit PAK1-4 in pharmacological inhibition/rescue experiments. FRAX486 was diluted in dimethyl sulphate (DMSO) to 10 mM per manufacturer's instructions. Differentiating hNPCs were treated with FRAX486 diluted in differentiation media 24 hours after transfection with HA-NLGN3/4X constructs at a final concentration of 50 nM (3, 4). DMSO was used as a vehicle treatment for control at the same dilution. FRAX486 and vehicle treated hNPCs continued differentiating for a further 48 hours in an incubator at 37°C/5% CO2 prior to fixation and immunocytochemistry.

***Microscopy***

Representative images of preliminary experiments in HEK293 and CTX0E16 cells, proliferating CTX0E16 experiments, mouse primary cortical neuron experiments, and all images for data analysis were acquired using a Zeiss Axio Imager Z1 epifluorescent microscope with ApoTome attachment using a 20x or 40x oil-immersion objective (Carl Zeiss AG). Representative images of further experiments investigating neurite outgrowth in differentiated CTX0E16 cells were acquired using a Leica SP-5 confocal microscope with a 100x oil-immersion objective (Leica microsystems). Z-stacks of 5 individual cells per condition across three biological replicates were

obtained for statistical data analysis. 10 images were taken per Z-stack at a slice distance of 0.5 μm.

Super-resolved structured illumination microscopy (SIM) images of growth cones were collected using a Visitech-iSIM module coupled to a Nikon Ti-E microscope with a Nikon 100x total internal reflection fluorescence (TIRF) oil immersion lens with a numerical aperture of 1.49 (Nikon, Japan). Blue fluorescence was excited with a 405 nm laser and emission filtered through a 460/50 filter. Green fluorescence was excited with a 488 nm laser and emission filtered through a 525/50 filter. Red Fluorescence was excited with a 561 nm laser and emission filtered through a 630/30 filter. Far Red fluorescence was excited with a 640 nm laser and emission filtered through a 710/60 filter. Multiple images at focal planes were collected spaced apart by 0.05 μm. Data were deconvolved using a Richardson-Lucy algorithm specific to the iSIM mode of imaging to increase contrast and resolution using the supplied NIS-Elements Advanced Research software (Nikon, Japan, v4.6).

***Quantification and Statistical Analysis***

Reads Per Kilobase of transcript per Million mapped reads (RPKM) values for NLGN3, NLGN4X, and ACTB expression were extracted from the publicly available BrainSpan Atlas of the Developing Human Brain compiled primarily by the Allen Institute for Brain Science (5). RPKM values were downloaded across multiple time points from all available donors spanning the earliest available time point (8 post-conception weeks) to the latest available time point in development (37 post-conception weeks). At each time point and for each available donor, RPKM values were extracted from all available brain regions including the hippocampus, amygdaloid complex, dorsolateral prefrontal cortex, ventrolateral prefrontal cortex, anterior (rostral) cingulate

(medial prefrontal) cortex, orbital frontal cortex, primary somatosensory cortex, posterior (caudal) superior temporal cortex, inferolateral temporal cortex, primary visual cortex, and the mediodorsal nucleus of thalamus. Raw RPKM values were further processed in MS Excel. Multiple values from the same donor at the same time point and for the same region were averaged to produce a single value. Data from each time point across all brain regions for NLGN3, NLGN4X, and ACTB were then averaged and plotted over time to produce an overall cortical expression level throughout human neurodevelopment.

All neurite outgrowth images were quantified using the NeuronJ ImageJ plug-in (http://www.imagescience.org/meijering/software/neuronj/ v1.4.2) which allowed for manual tracing and labelling (primary, secondary, or tertiary and axon/dendrite) of individual neurites (6) **(Fig S7C)**. Neurite inclusion/exclusion criteria were established visually while tracing and later re-established once all neurites were traced via a $<3$ μm neurite length exclusion threshold set via logic test in MS Excel. Five cells per condition were quantified across three biological replicates.

Growth cone area was quantified using NIS-Elements Advanced Research software (Nikon, Japan). Growth cone filament number was quantified using line scan analysis in ImageJ **(Fig S7D-E)**. Growth cone filament skeletons were generated using the ImageJ plug-in Ridge Detection (7). Filament length was also quantified using Ridge Detection. Growth cone filament anisotropy was quantified using the ImageJ plug-in FibrilTool (8).

All immunoblotting and RT-qPCR data were processed post-hoc in MS Excel to eliminate batch effects (9). Briefly, the sum of all conditions in each data set was calculated. Each condition was then divided by the sum of all conditions to remove batch effects. Data were then log transformed and plotted. All datasets were tested for normality using the D'Agostino & Pearson normality test prior to inferential statistical analyses (10). Datasets found to be normally distributed

were analysed using parametric statistical tests while datasets found to be abnormally distributed were analysed using their non-parametric equivalent. Two-tailed unpaired students t-test or Mann Whitney U test was used for endogenous expression protein/RNA analyses with an alpha level of 0.05. Ordinary one-way analysis of variance (ANOVA) with Bonferroni correction for multiple comparisons or Kruskal-Wallis test with Dunn's post-hoc correction was used for total neurite outgrowth analyses and axon/dendrite analyses with an alpha level of 0.05 (MRD, Mean Rank Difference). Two-way ANOVA with Bonferroni correction for multiple comparisons was used for primary, secondary, and tertiary neurite outgrowth analyses and axon/dendrite analyses. Ordinary one-way ANOVA with Bonferroni correction for multiple comparisons or Kruskal-Wallis test with Dunn's post-hoc correction was used for all super-resolution growth cone actin analyses with an alpha level of 0.05. One-way ANOVA with Bonferroni correction for multiple comparisons was used for all immunoblotting data analyses with an alpha level of 0.05. Two-way ANOVA with Bonferroni correction for multiple comparisons was used for FRAX486 growth cone actin filament attenuation analyses with an alpha level of 0.05. Two-tailed unpaired students t-test or Mann Whitney U test was used for FRAX486 treated growth cone NLGN cluster analyses. Lastly, two-way ANOVA with Bonferroni correction for multiple comparisons was used for FRAX486 treated neurite outgrowth analyses. All data visualisations were generated in GraphPad Prism 8.0 (GraphPad Software, La Jolla California USA, http://www.graphpad.com/scientific-software/prism/). All data are shown as mean ± standard error of the mean (SEM) to two decimal places where necessary and all error bars represent SEM. Full reports of all statistical results are presented in **Table S3,** all data from the main figures are presented in **Table S4,** and all data from the supplemental figures are presented in **Table S5.**

SUPPLEMENTAL FIGURES

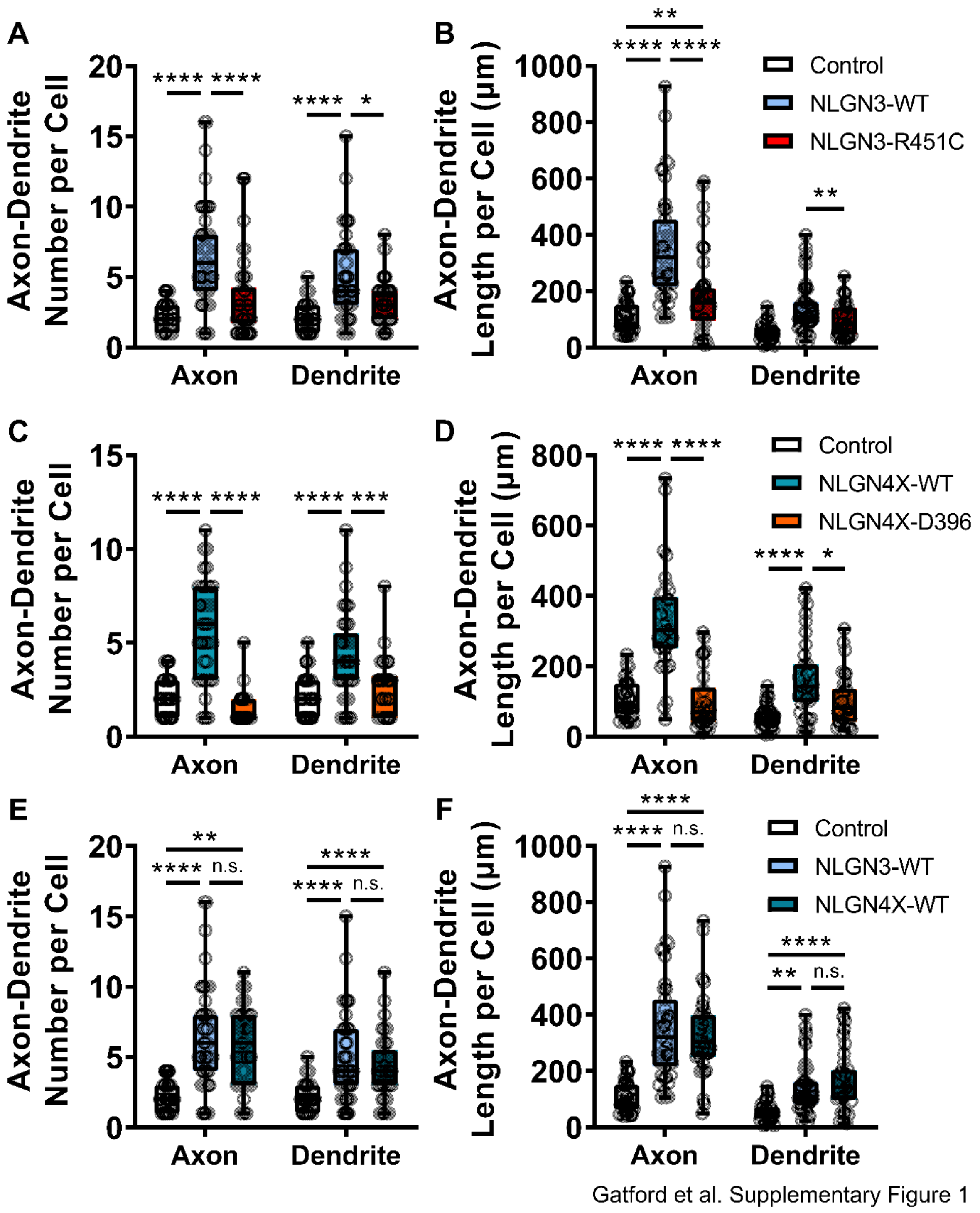


**Figure S1. NLGN3/4X-WT promote axon and dendrite outgrowth in mouse primary cortical neurons. (A)** Data showing ectopically expressed NLGN3-WT promotes axon and dendrite

number in mouse primary cortical neurons. **(B)** Data showing ectopically expressed NLGN3-WT promotes axon and dendrite length while NLGN3-R451C increases axon length in mouse primary cortical neurons. **(C)** Data showing ectopically expressed NLGN4X-WT promotes axon and dendrite number in mouse primary cortical neurons. **(D)** Data showing ectopically expressed NLGN4X-WT promotes axon and dendrite length in mouse primary cortical neurons. **(E)** Data showing ectopically expressed NLGN3-WT and NLGN4X-WT do not differentially influence axon or dendrite number in mouse primary cortical neurons. **(F)** Data showing ectopically expressed NLGN3-WT and NLGN4X-WT do not differentially influence axon or dendrite length in mouse primary cortical neurons. All significance was confirmed by parametric one-way ANOVA with Bonferroni post-hoc correction or non-parametric Kruskal-Wallis H test with Dunn's post-hoc test.

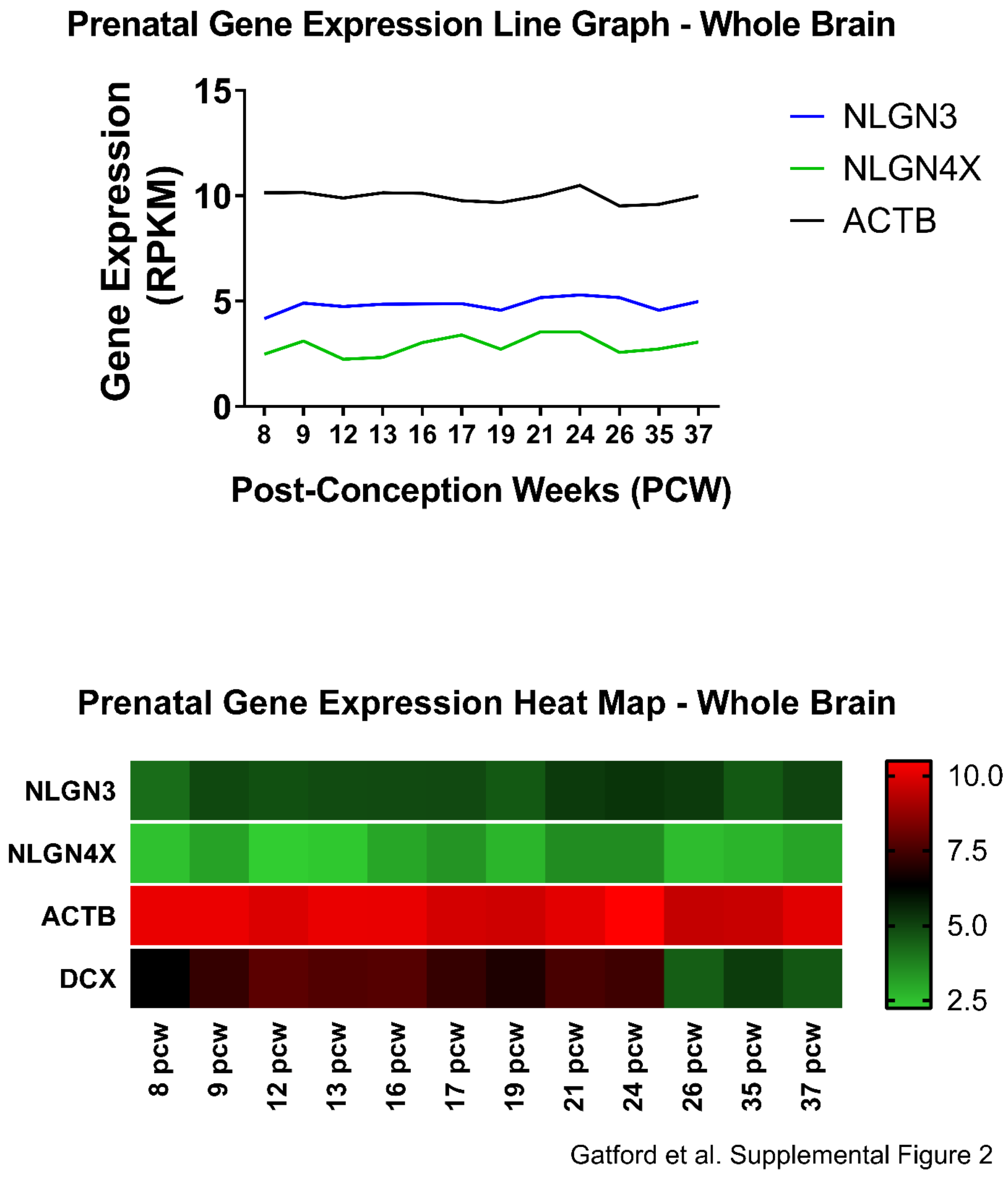


**Figure S2. Prenatal gene expression data reveals NLGN3 and NLGN4X are expressed during human prenatal neurodevelopment. (A)** Prenatal human gene expression of NLGN3 and

NLGN4X compared to beta-actin (ACTB) compiled from the Allen Brain Atlas shown as a line graph. **(B)** Prenatal human gene expression of NLGN3 and NLGN4X compared to beta-actin (ACTB) and doublecortin (DCX) compiled from the Allen Brain Atlas shown as a heat map.

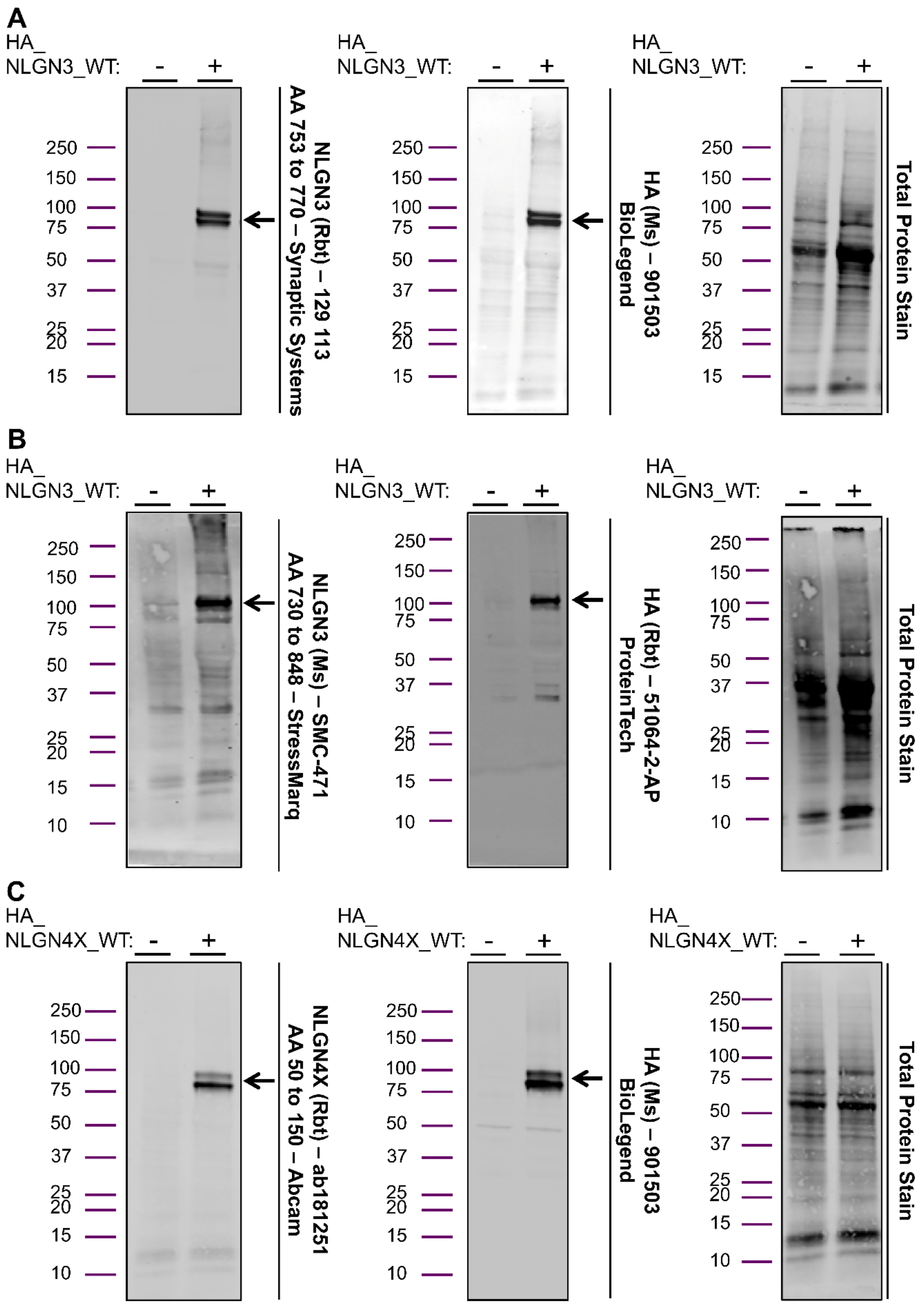


Gatford et al. Supplemental Figure 3

**Figure S3. NLGN3 and NLGN4X antibodies are highly specific (A)** Representative western blots demonstrating the NLGN3 (Rabbit) antibody to NLGN3 AA 753 to 770 is highly specific to NLGN3 and the HA (Mouse) antibody is highly specific to HA. **(B)** Representative western blots demonstrating the NLGN3 (Mouse) antibody to NLGN3 AA 730 to 848 is also highly specific to NLGN3 and the HA (Rabbit) antibody is highly specific to HA. **(C)** Representative western blots demonstrating the NLGN4X (Rabbit) antibody to NLGN4X AA 50 150 is highly specific to NLGN4X.

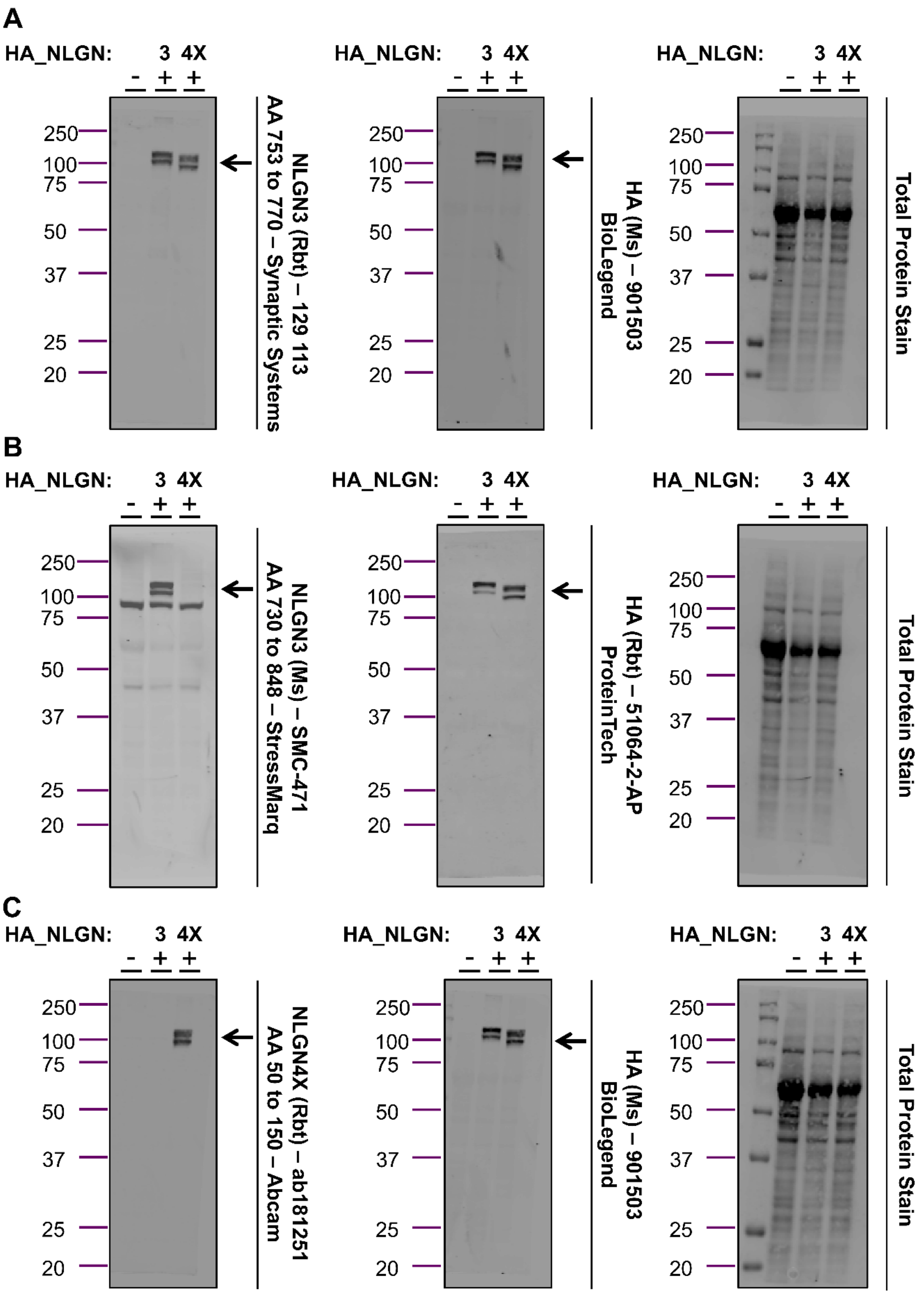


Gatford et al. Supplemental Figure 4

**Figure S4. NLGN3 and NLGN4X antibodies have a degree of cross reactivity. (A)** Representative western blots showing cross reactivity for the NLGN3 (Rabbit) antibody to NLGN3 AA 753 to 770 with NLGN4X. **(B)** Representative western blots showing cross reactivity for the NLGN3 (Mouse) antibody to NLGN3 AA 730 to 848 with NLGN4X. **(C)** Representative western blots showing cross reactivity for the NLGN4X (Rabbit) antibody to NLGN4X AA 50 to 150 with NLGN

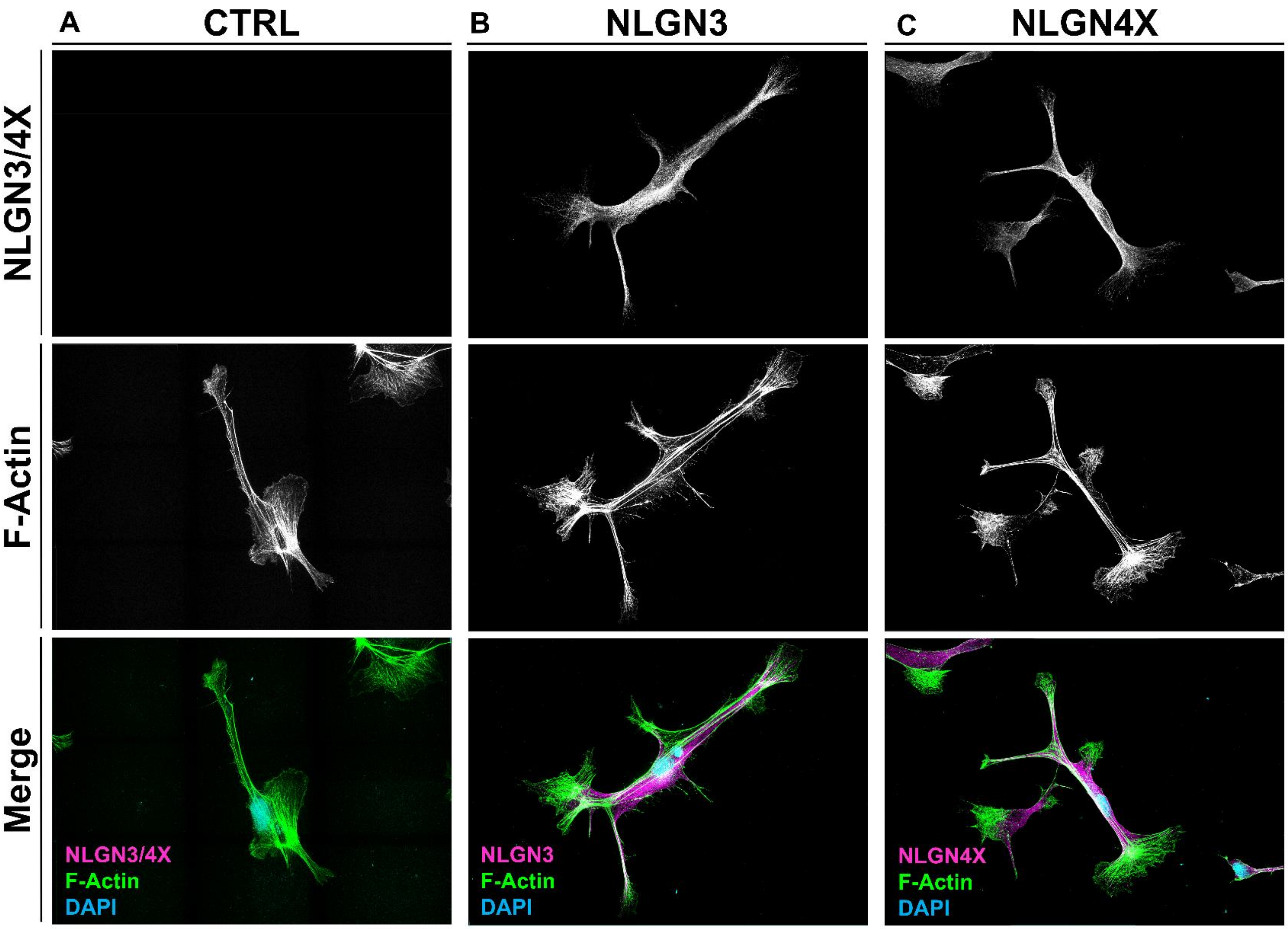


Gatford et al. Supplemental Figure 5

**Figure S5. Ectopic NLGN3 and NLGN4X localises throughout the cell.** Representative super-resolution images of differentiating CTX0E16 immature human neurons showing ectopic wildtype NLGN3/4X protein localises to the cell soma, neurites, and extremities. Scale bar = 25μm (full cell), 5μm (magnified).

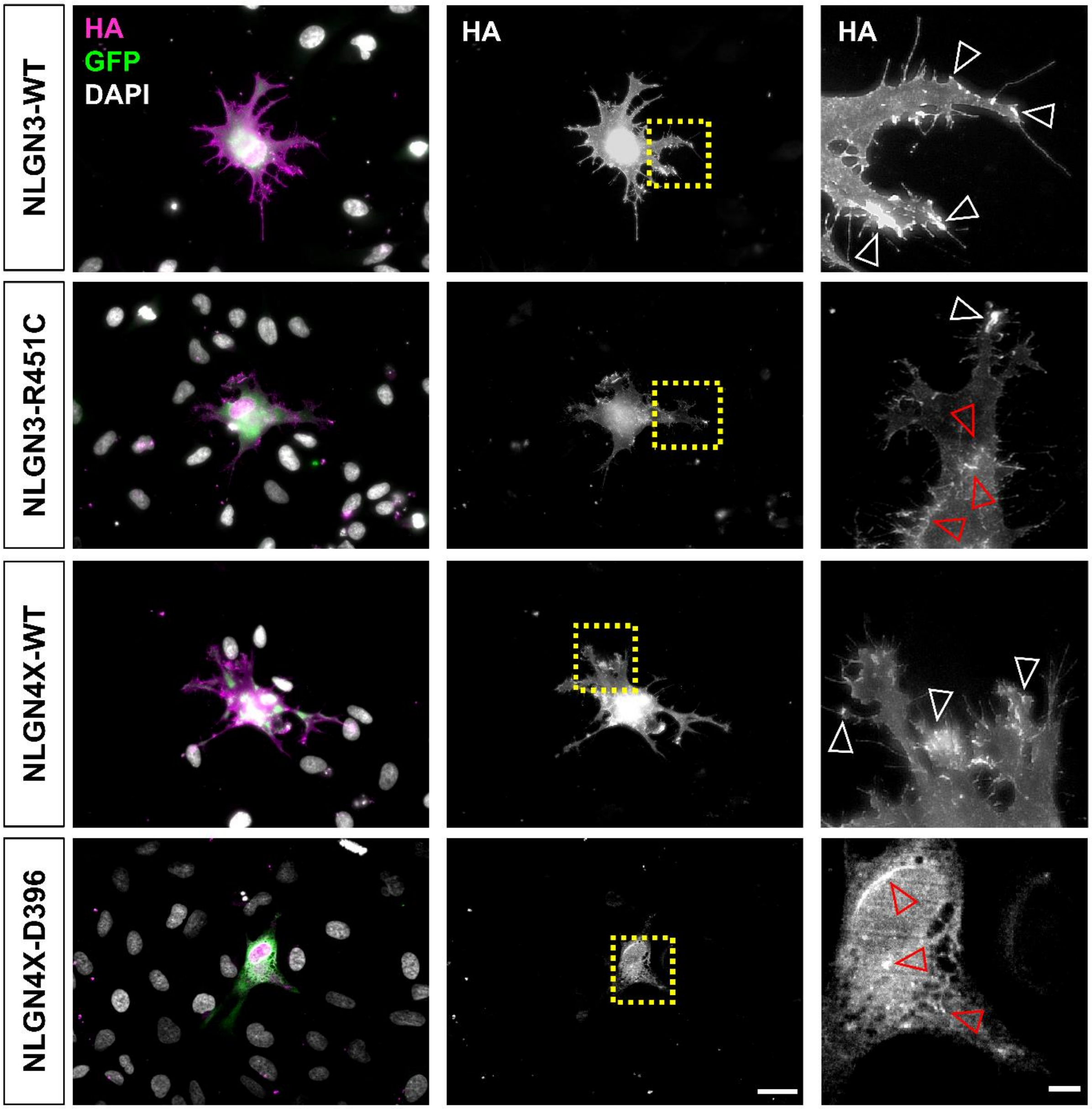


Gatford et al. Supplemental Figure 6

**Figure S6. NLGN3 and NLGN4X localise at the tips of protrusions while mutant variants mislocalise.** Representative epifluorescent images of CTX0E16 human neural progenitor cells showing wildtype NLGN3/4X protein localising in growth cones of protrusions (white open arrows) while mutant variant proteins mislocalise to the cytosol (red open arrows). Scale bar = 25µm (full cell), 5µm (magnified).

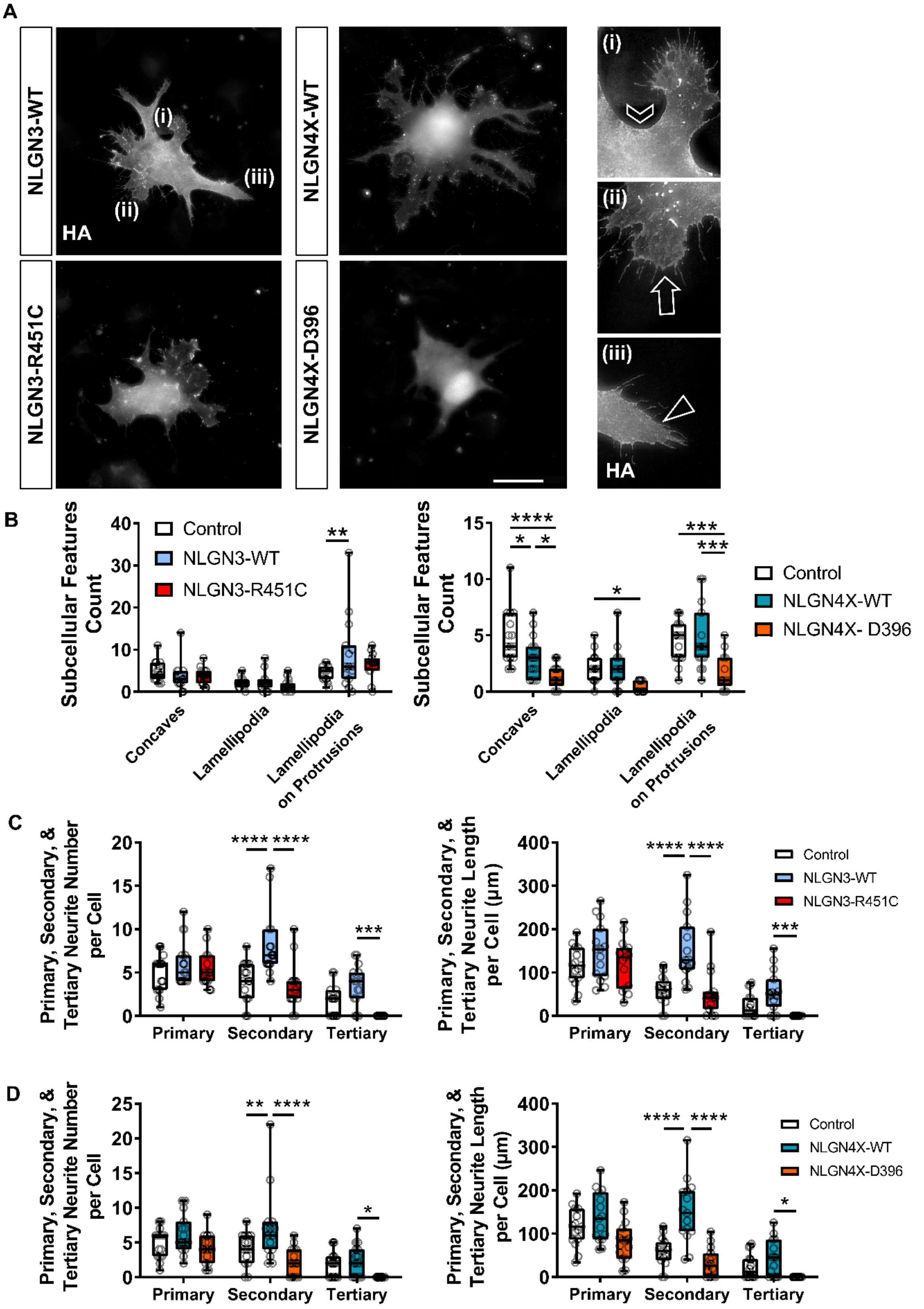


Gatford et al. Supplemental Figure 7

13

**Figure S7. NLGN3/4X-WT and their mutant forms exert differential effects on subcellular features and neurites. (A)** Representative images showing ectopic NLGN3 and NLGN4X expression in human neural progenitor cells induces cell membrane structural changes which are not induced by ectopic expression of their mutant variants in human neural progenitor cells. Chevron = concave, arrow = lamellipodia, triangle = lamellipodia on protrusion. Scale bar = 25µm. **(B)** Data showing ectopic NLGN3 expression significantly increases the number of lamellipodia on protrusions, confirmed by parametric one-way ANOVA with Bonferroni post-hoc correction. **(C)** Data showing ectopic NLGN3-WT expression significantly increases secondary neurite length and number in immature human neurons, confirmed by parametric two-way ANOVA with Bonferroni post-hoc correction. **(D)** Data showing ectopic NLGN4X-WT expression significantly increases primary neurite length as well as secondary neurite length and number in immature human neurons, confirmed by parametric two-way ANOVA with Bonferroni post-hoc correction.

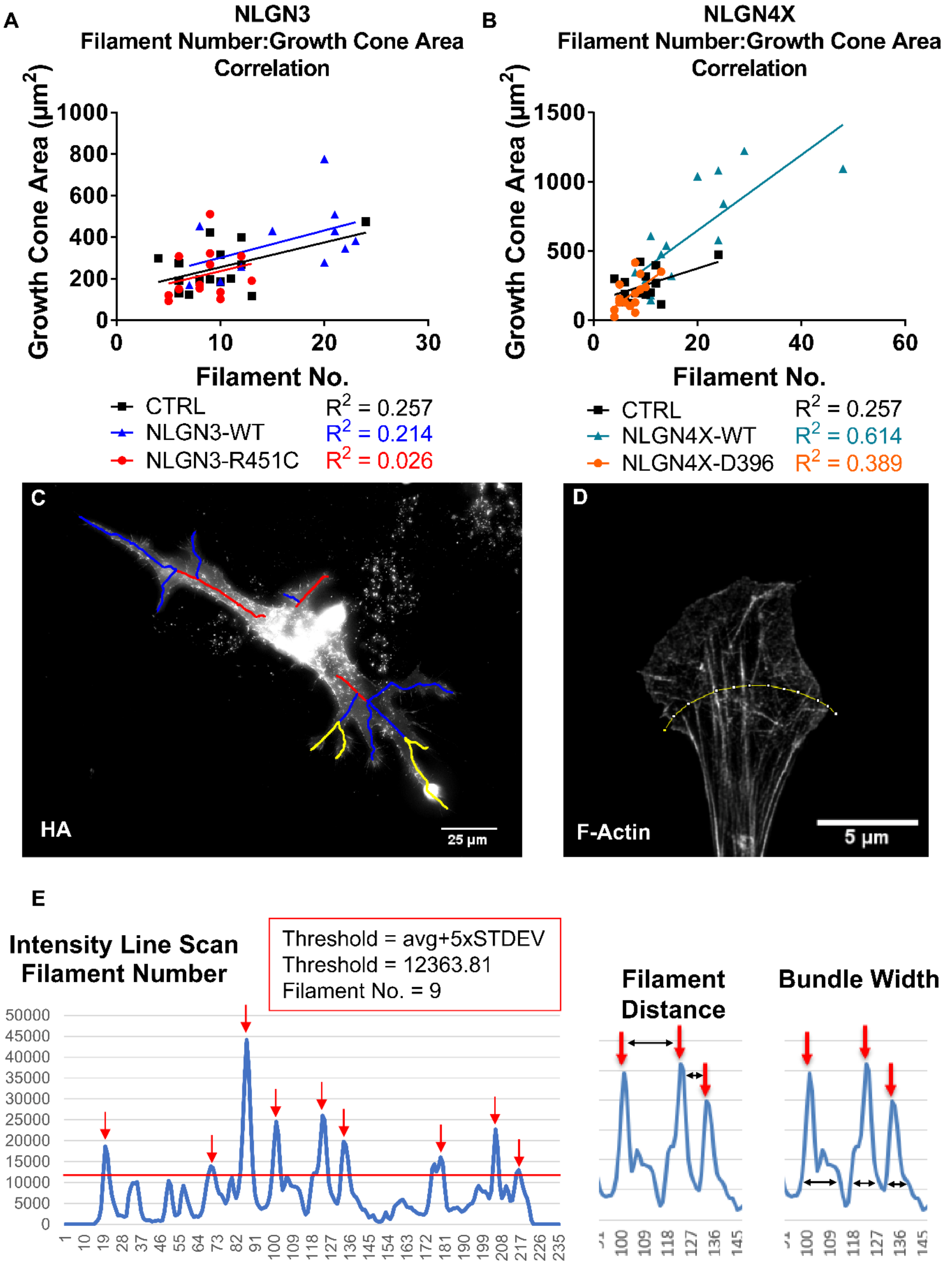


Gatford et al. Supplementary Figure 8

27

**Figure S8. Filament number is correlated to growth cone area and the methodology used to quantify filaments. (A)** Correlation data showing filament number and growth cone area are correlated in growth cones ectopically expressing wildtype NLGN3 and NLGN3-R451C. **(B)** Correlation data showing filament number and growth cone area are correlated in growth cones ectopically expressing wildtype NLGN4X and NLGN4X-D396. **(C)** Example image of a CTX0E16 human neural progenitor cell ectopically expressing wildtype NLGN3 with overlaid neurite tracings; red, primary neurite; blue, secondary neurite; yellow, tertiary neurite. Scale bar = 25µm. **(D)** Example image of F-actin in a growth cone from an untransfected control CTX0E16 immature neuron with a line scan overlay showing how actin filaments were detected. Scale bar = 5µm. **(E)** Example graphs showing data output from the line scan in (**Supplementary Fig 7D**), illustrating how the background threshold was calculated, how filaments were counted, how filament distance was calculated, and how bundle width was measured.

Gatford et al. Supplemental Figure 9

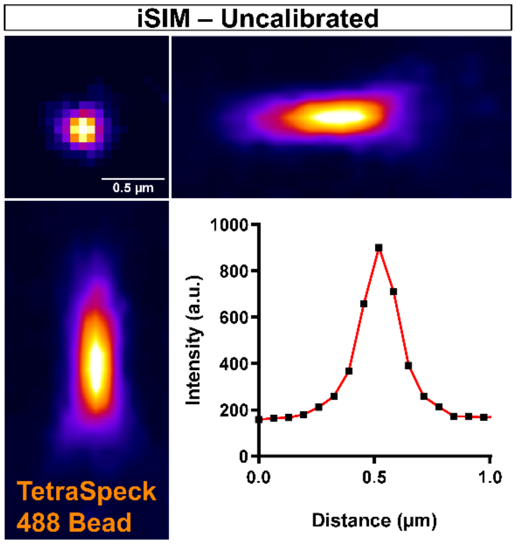


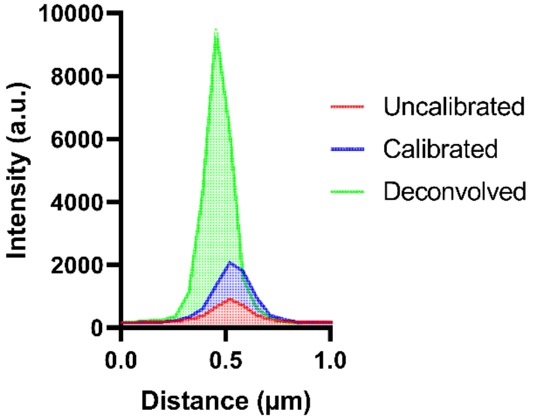


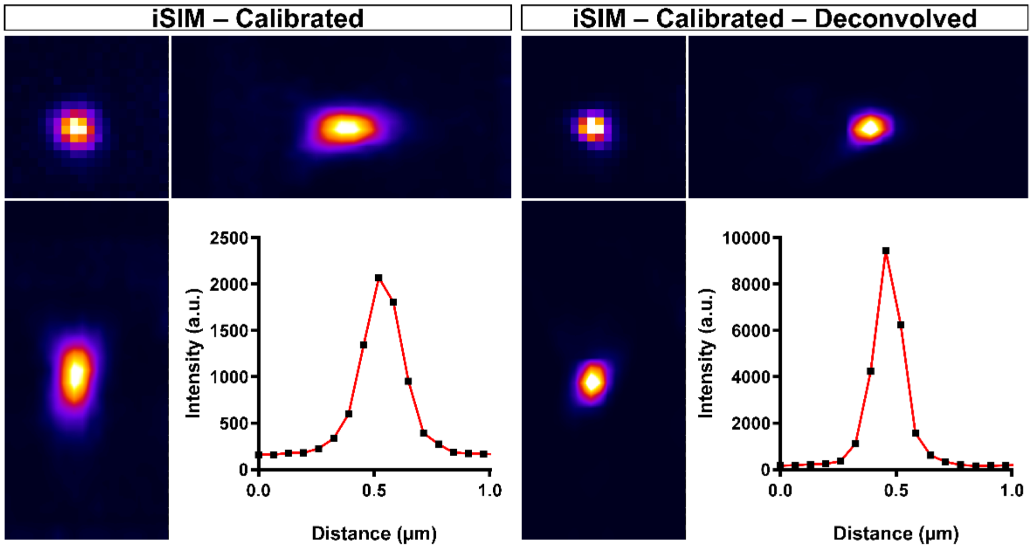


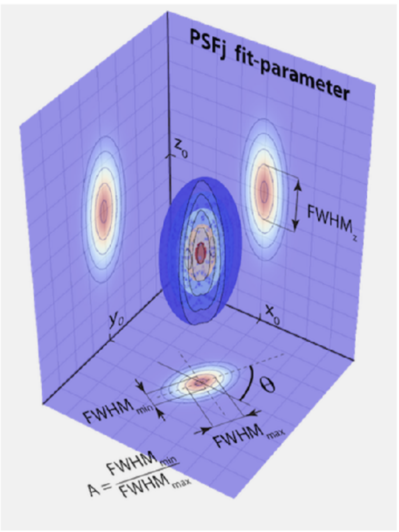


| Lens | FW HM | Bead Size (nm) | Theoretical (nm) | Asymmetry (A) |
|---|---|---|---|---|
| 100x oil | Min | 196 | 181 | 0.841 |
| | Max | 233 | 181 | |
| | Z | 400 | 633 | |
| 100x oil (Deconvolved) | Min | 161±5 | 181 | 0.834±0.027 |
| | Max | 194±7 | 181 | |
| | Z | 366±8 | 633 | |
| 100x Silicon | Min | 199±35 | 199 | 0.871±0.091 |
| | Max | 235±72 | 199 | |
| | Z | 449±120 | 713 | |
| 100x Silicon (Deconvolved) | Min | 140±24 | 199 | 0.878±0.084 |
| | Max | 163±50 | 199 | |
| | Z | 338±37 | 713 | |

**Figure S9. Nikon iSIM calibration data.** Representative images of a single TetraSpeck fluorescent bead imaged using a 100x TIRF lens and Nikon iSIM; including uncalibrated (upper left), calibrated (lower left), and calibrated/deconvolved (lower right). Scale bar = 0.5 μm. Data table shows full width at half maximum (FWHM) measurements averaged across multiple beads in a single field of view compared to their theoretical measurements and their calculated asymmetry. PSF diagram adapted from PSFj ImageJ plug-in documentation (Theer et al., 2014).

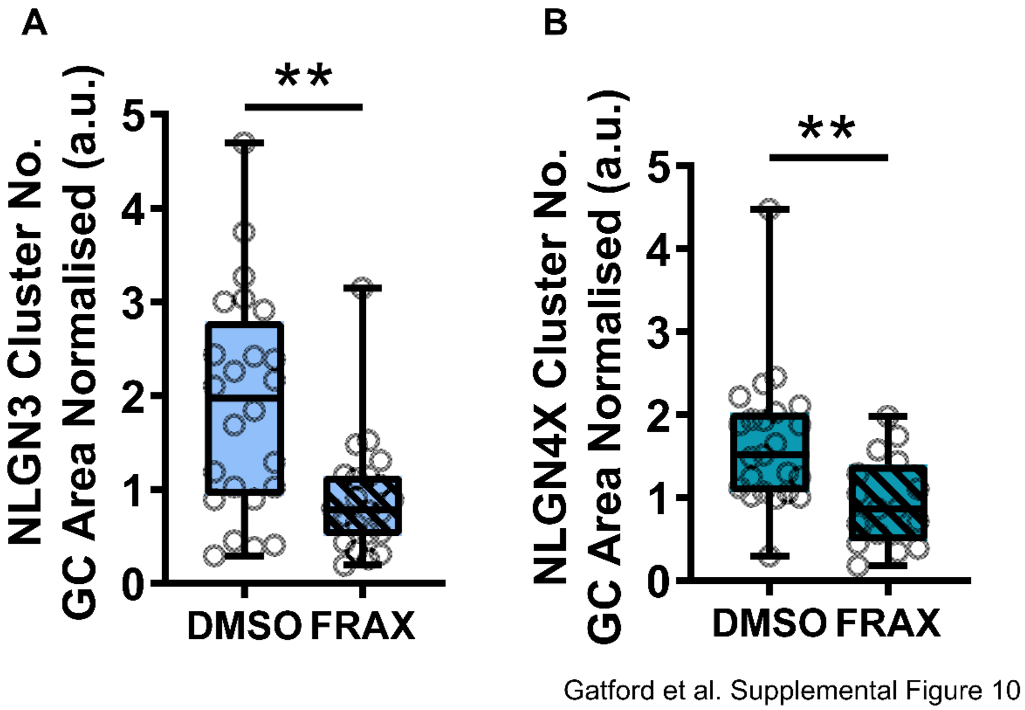


**Figure S10. Growth cone NLGN3/4X cluster number remains decreased when normalised to growth cone area. (A)** Data showing ectopically expressed HA-NLGN3-WT clusters still decrease when treated with FRAX486 compared to untreated when normalised to growth cone area. **(B)** Data showing ectopically expressed HA-NLGN4X-WT clusters still decrease when treated with FRAX486 compared to untreated when normalised to growth cone area.

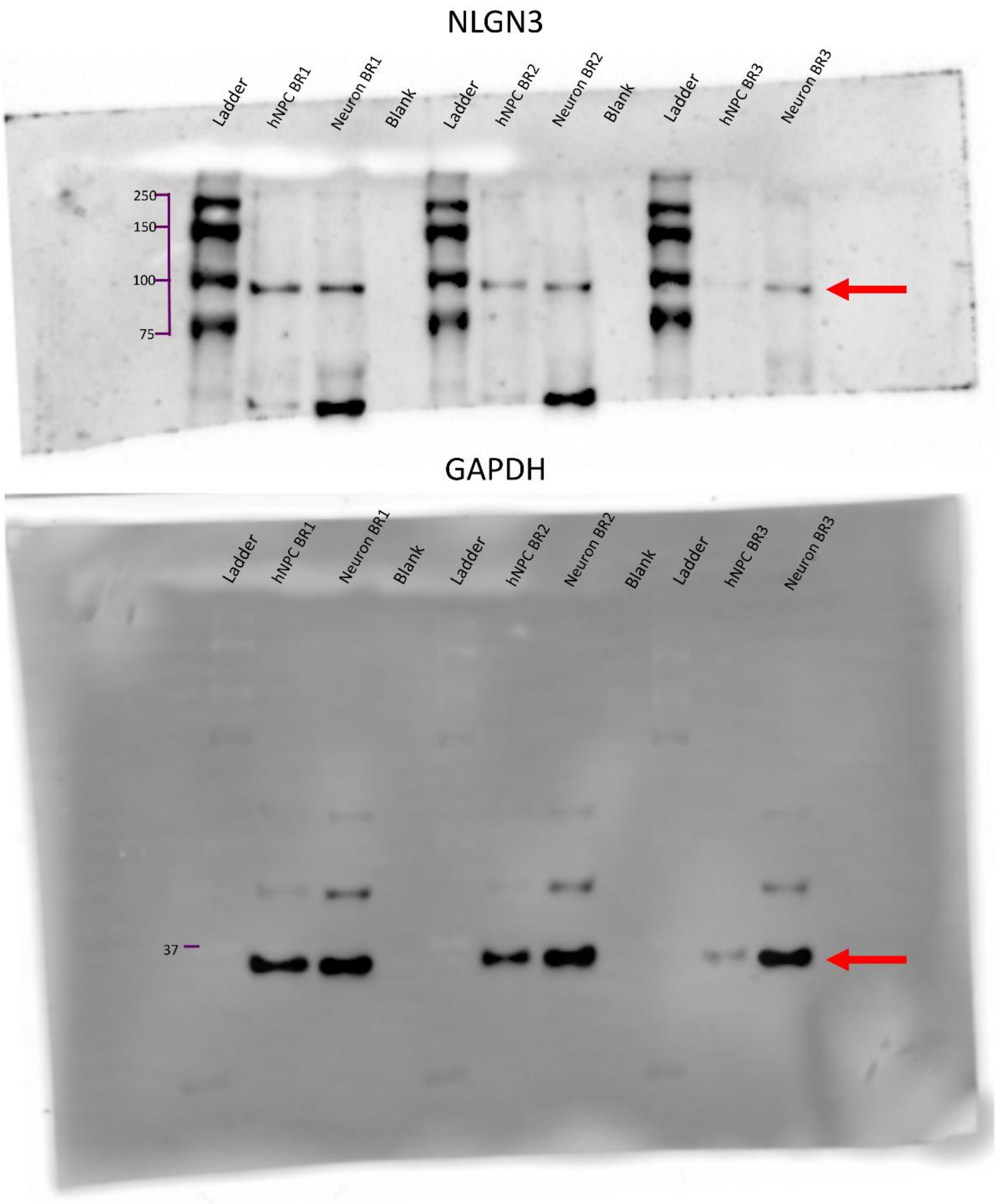


Full western blot images for Figure 3B – NLGN3. Red arrow indicates the quantified band across all lanes. hNPC – human neural progenitor cell, BR – biological replicate, NLGN3 – Neuroligin-3, GAPDH – Glyceraldehyde 3-phosphate dehydrogenase.

NLGN4X

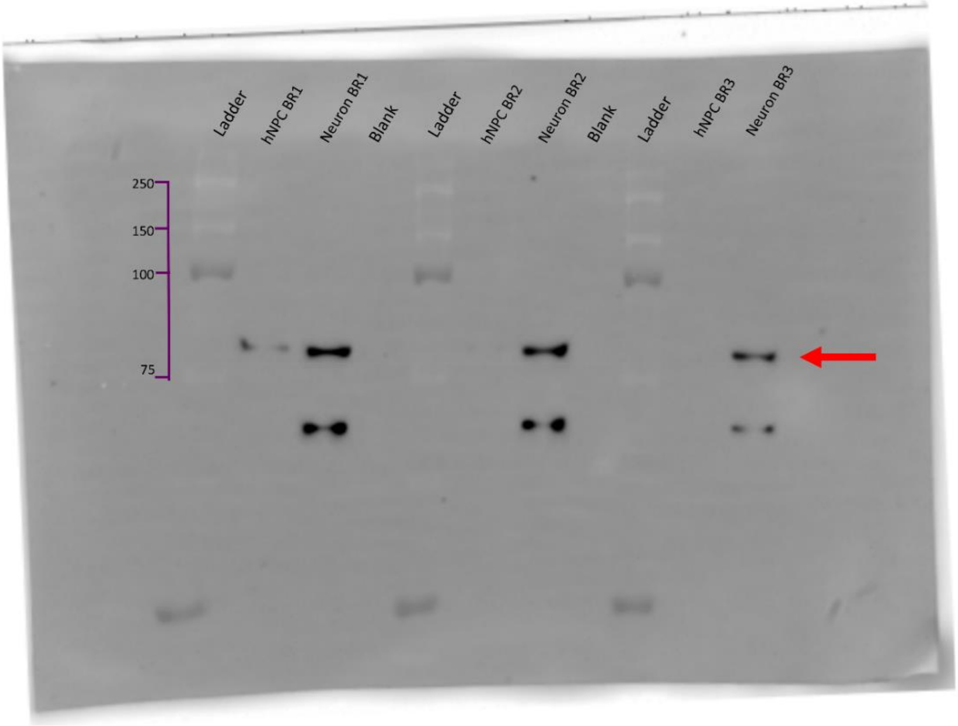


GAPDH

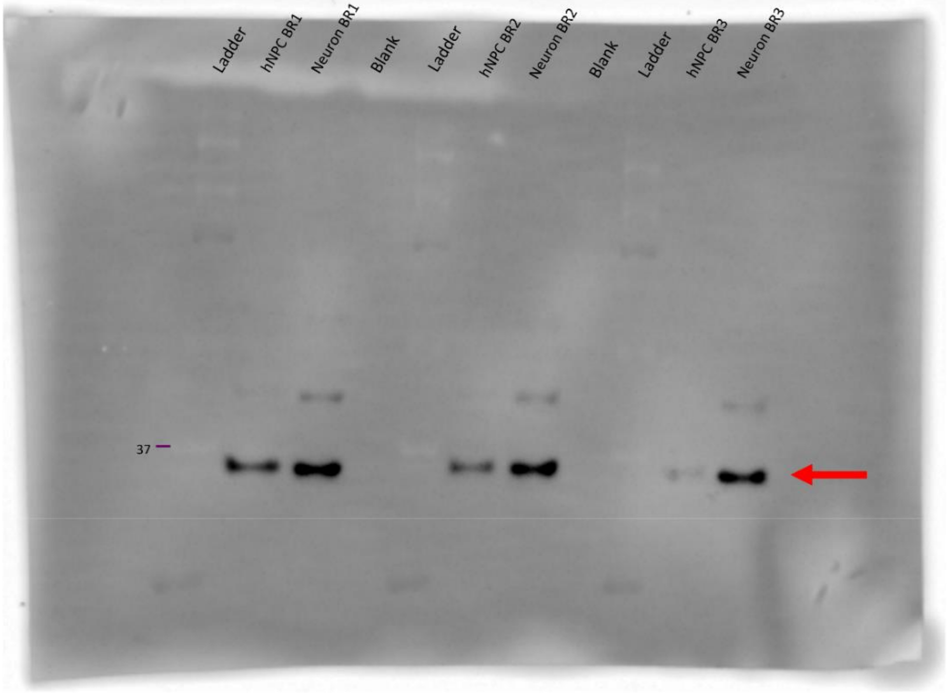


59

Full western blot images for Figure 3B – NLGN4X. Red arrow indicates the quantified band across all lanes. hNPC – human neural progenitor cell, BR – biological replicate, NLGN4X – Neuroligin-4X, GAPDH – Glyceraldehyde 3-phosphate dehydrogenase.

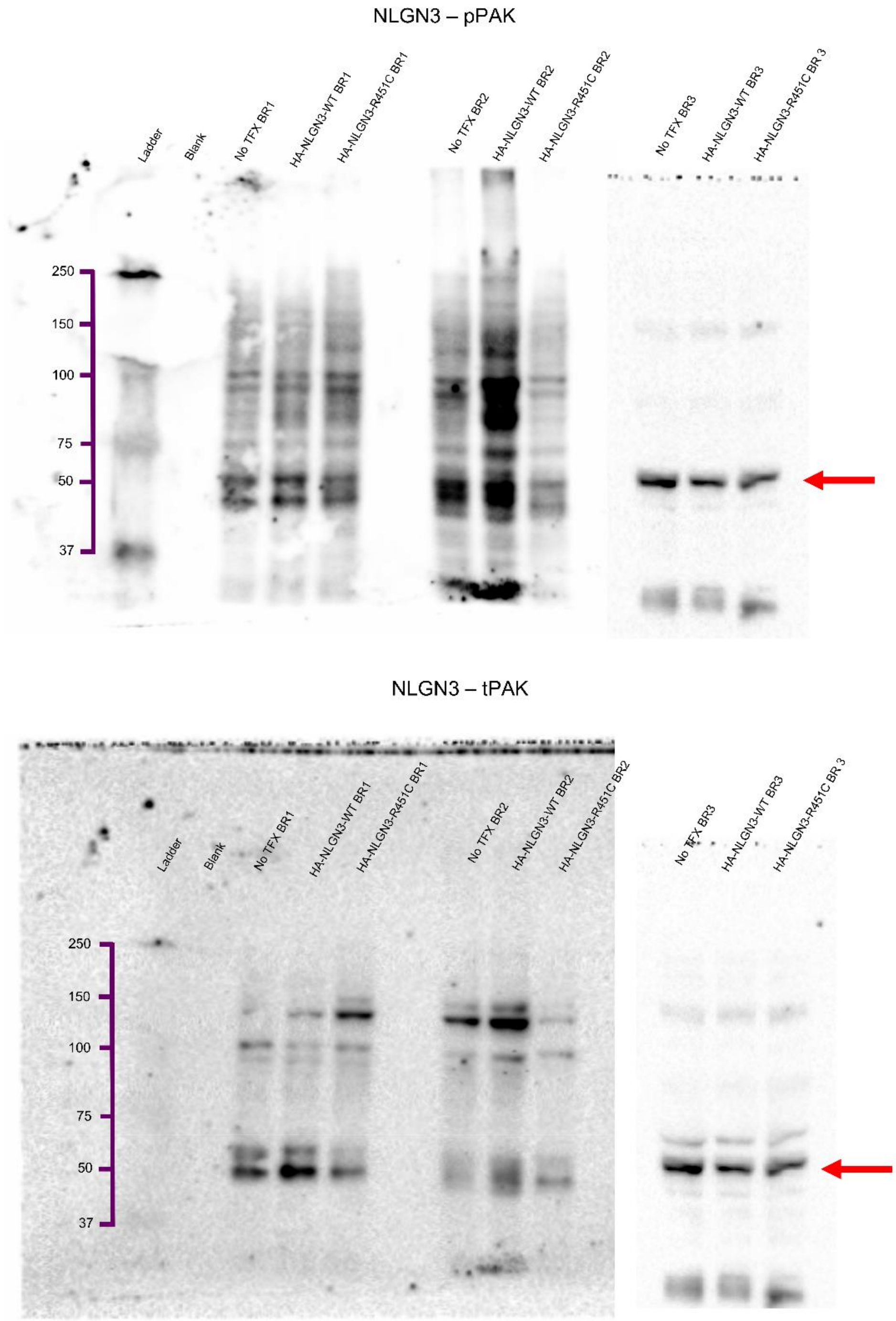


Full western blot images for Figure 6A. Red arrow indicates the quantified band across all lanes. p/tPAK1 – total/phospho-p21-activated kinase, BR – biological replicate, NLGN3 – Neuroligin-3, TFX – Transfection, HA – Haemagglutinin.

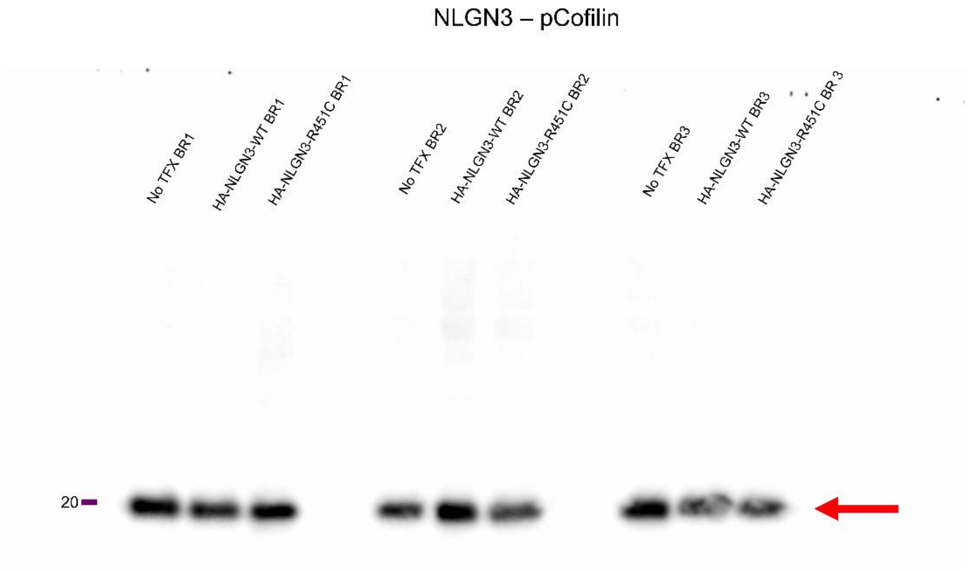


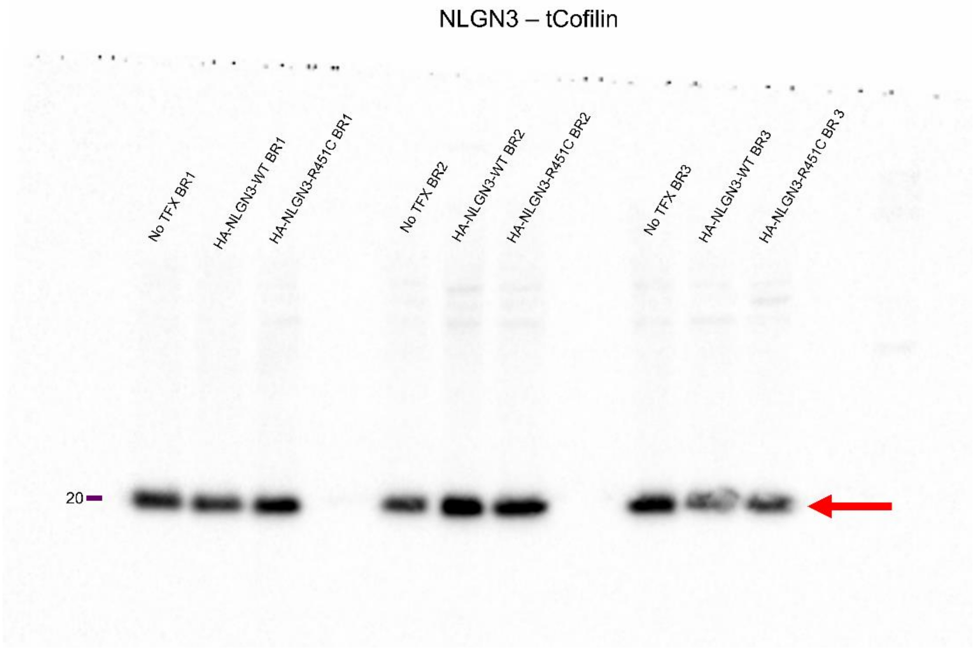


Full western blot images for Figure 6A. Red arrow indicates the quantified band across all lanes. t/p – total/phospho, BR – biological replicate, NLGN3 – Neuroligin-3, TFX – Transfection, HA – Haemagglutinin.

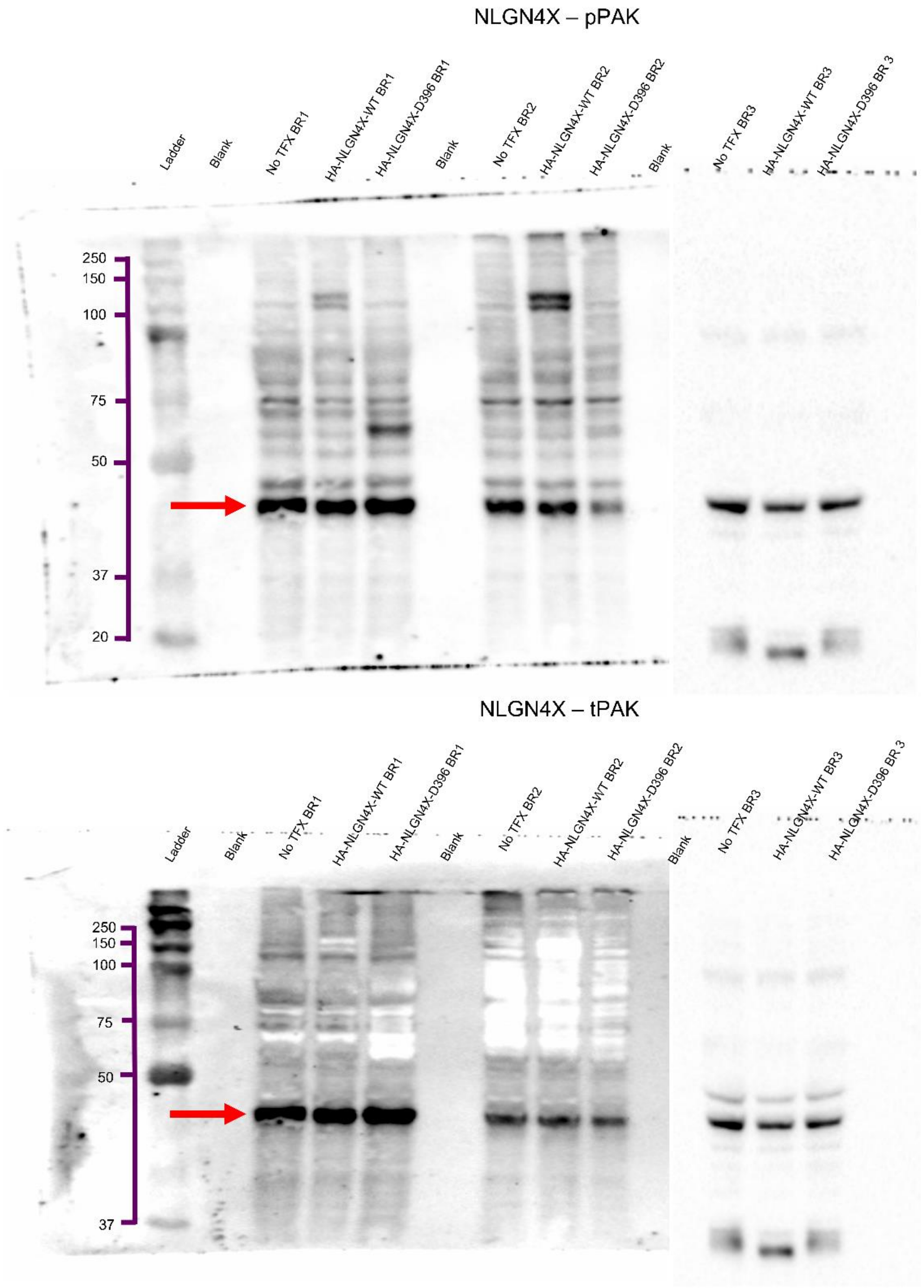


Full western blot images for Figure 6B. Red arrow indicates the quantified band across all lanes. p/tPAK1 – total/phospho-p21-activated kinase, BR – biological replicate, NLGN4X – Neuroligin-4X, TFX – Transfection, HA – Haemagglutinin.

NLGN4X – pCofilin

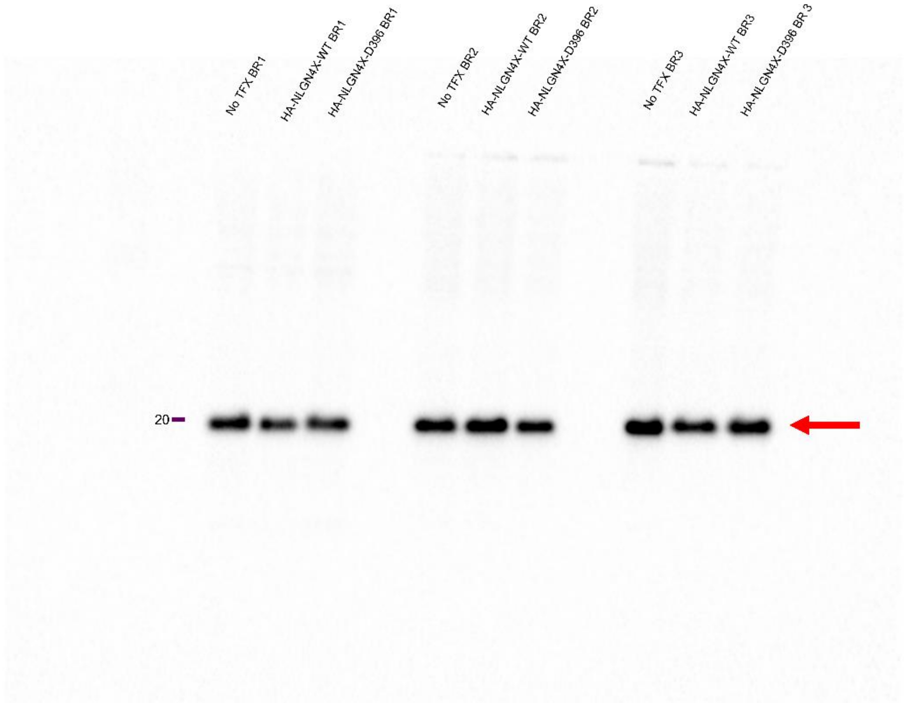


NLGN4X – pCofilin

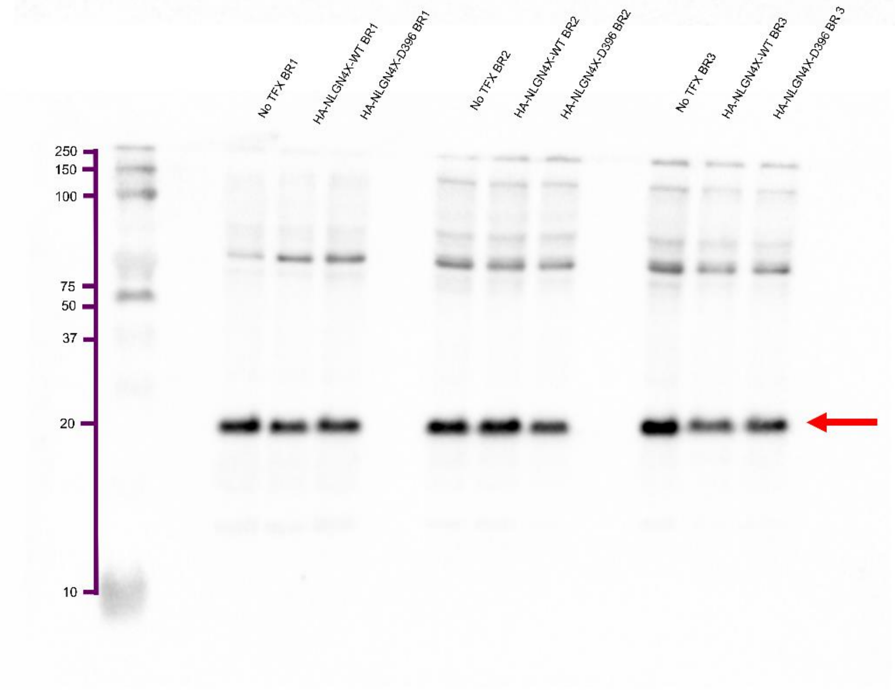


76

Full western blot images for Figure 6B. Red arrow indicates the quantified band across all lanes. t/p – total/phospho, BR – biological replicate, NLGN4X – Neuroligin-4X, TFX – Transfection, HA – Haemagglutinin.

## SUPPLEMENTAL TABLES

| Epitope | Species | Clonality | Company | Clone | Cat. # | Lot # | Conc. (ICC) | Conc. (WB) |
|---|---|---|---|---|---|---|---|---|
| **HA** | Ms | mAb | BioLegend | 16B12 | 901503 | B198897 | 1:1000 | 1:2000 |
| **HA** | Rbt | pAb | ProteinTech | N/A | 51064-2-AP | 00046618 | N/A | 1:5000 |
| **NLGN3** | Ms | mAb | StressMarq | S110-29 | SMC-471 | 130924 | 1:1000 | 1:500 |
| **NLGN3** | Rbt | pAb | Synaptic Systems | N/A | 129 113 | 129113/5 | 1:1000 | 1:500 |
| **NLGN4X** | Rbt | mAb | Abcam | EPR13108 | ab181251 | GR162222-1 | 1:1000 | 1:1000 |
| **Myc** | Ms | mAb | BioLegend | 9E10 | 626801 | B222211 | N/A | 1:200 |
| **GFP** | Chk | pAb | Abcam | N/A | ab13970 | GR53074 | 1:1000 | N/A |
| **RFP** | Rbt | pAb | MBL | N/A | PM005 | 042 | 1:500 | N/A |
| **Tuj1** | Chk | pAb | Abcam | N/A | ab41489 | GR118611 | 1:1000 | N/A |
| **MAP2** | Rbt | pAb | Abcam | N/A | ab32454 | GR3190640 | 1:1000 | N/A |
| **Phalloidin Actin Green 488** | N/A | N/A | Molecular Probes | N/A | R37110 | 1892114, 1924467, 1941122 | N/A | N/A |
| **Phospho-PAK1** | Rbt | pAb | Cell Signaling Technology | N/A | 2606 | 6 | 1:200 | 1:1000 |
| **Total-PAK1** | Rbt | pAb | Cell Signaling Technology | N/A | 2604 | 2 | N/A | 1:1000 |
| **Phospho-Cofilin** | Rbt | mAb | Cell Signaling Technology | 77G2 | 3313 | 7 | N/A | 1:1000 |
| **Total-Cofilin** | Ms | mAb | ProteinTech | 1G6A2 | 66057-1-lg | 1000917 | N/A | 1:3000 |
| **Alexa Fluor 488** | Goat Anti-Ms | pAb | Life Technologies | N/A | A-21050 | SG251135 | 1:500 | N/A |
| **Alexa Fluor 568** | Goat Anti-Rbt | pAb | Life Technologies | N/A | A-11063 | 1924788 | 1:500 | N/A |
| **Alexa Fluor 633** | Goat Anti-Chk | pAb | Life Technologies | N/A | A-11039 | 1806122 | 1:500 | N/A |

| | | | | | | | | |
|---|---|---|---|---|---|---|---|---|
| **Alexa Fluor 680** | Goat Anti-Ms | pAb | Thermo Fisher | N/A | A21058 | 1600888 | N/A | 1:500 |
| **Alexa Fluor 790** | Goat Anti-Rbt | pAb | Thermo Fisher | N/A | A11367 | 1152220 | N/A | 1:500 |
| **DAPI** | N/A | N/A | Molecular Probes | N/A | D1306 | 1503621 | 1:50000 | N/A |
| **HRP** | Rbt | pAb | Invitrogen | N/A | 31460 | SH253595 | N/A | 1:10000 |
| **HRP** | Ms | pAb | Invitrogen | N/A | 31430 | SH252846 | N/A | 1:10000 |

**Key**: HA – Haemagglutinin, NLGN – Neuroligin, GFP – Green fluorescent protein, RFP – Red fluorescent protein, Tuj1 – Beta-III-Tubulin, MAP2 – Microtubule Associated Protein 2, PAK – p21-activated kinase, DAPI – 4′,6-diamidino-2-phenylindole, HRP – Horseradish peroxidase, Ms – Mouse, Rbt – Rabbit, Chk – Chicken, mAb – Monoclonal, pAb – Polyclonal, Cat. – Catalogue, Conc. – Concentration, ICC – Immunocytochemistry, WB – Western blot

Gatford et al. Table S1

| Gene | Sequence (5’>3’) | Use | Company |
|---|---|---|---|
| **NLGN3_F** | CAG GGA GGC TCT TAC ATG GA | RT-qPCR | IDT |
| **NLGN3_R** | AGC ACT CCA ACC CGA TAG TT | RT-qPCR | IDT |
| **NLGN4X_F** | TGT TCT TAG GCC CCG GAT TT | RT-qPCR | IDT |
| **NLGN4X_R** | CTC CAA TGT CCT CCT GTG GT | RT-qPCR | IDT |
| **ALG2_F** | ACA ACT GAG TCA AAG CTG GTG | RT-qPCR, HK Screen | IDT |
| **ALG2_R** | GGA AAC ATT CAA GTC CCT GTC T | RT-qPCR, HK Screen | IDT |
| **RPL6_F** | CAG AGC AAA CAC AGA TCG CA | RT-qPCR, HK Screen | IDT |
| **RPL6_R** | ATG AGA TTA CGG AGC AGC GC | RT-qPCR, HK Screen | IDT |
| **HRH1_F** | TAT CGT ACC AAG ACC CGA GC | HK Screen | IDT |
| **HRH1_R** | GAG GTC TGC TGC ATG AAG TG | HK Screen | IDT |
| **ATE1_F** | ACC GTA GTG CAG AGT AGA CG | HK Screen | IDT |
| **ATE1_R** | AGT ACT GGC TTG ACG GAA AGA | HK Screen | IDT |

**Table S2** – A table detailing the forward and reverse primer sequences used in this study. IDT, Integrated DNA Technologies; HK, Housekeeper; ALG2, Asparagine-Linked Glycosylation 2 Homolog; RPL6, Ribosomal Protein L6; ATE1, Arginyltransferase 1.

Gatford et al. Table S2

| Figure | Variable | Conditions | ND? | Test | Post-hoc | Test Stat | MC | df | P value (sig) | Adj P value (sig) | n |
|---|---|---|---|---|---|---|---|---|---|---|---|
| 1B – NLGN3 | Neurite count/cell | CTRL – NLGN3-WT | N | Kruskal-Wallis | Dunn MRD | 50.80 | -55.49 | 3 | <0.0001 (****) | <0.0001 (****) | 30 |
| 1B – NLGN3 | Neurite count/cell | CTRL – NLGN3-R451C | N | Kruskal-Wallis | Dunn MRD | 50.80 | -21.49 | 3 | <0.0001 (****) | 0.02 (*) | 30 |
| 1C – NLGN3 | Neurite length/cell | CTRL – NLGN3-WT | N | Kruskal-Wallis | Dunn MRD | 57.80 | -59.96 | 3 | <0.0001 (****) | <0.0001 (****) | 30 |
| 1C – NLGN3 | Neurite length/cell | CTRL – NLGN3-R451C | N | Kruskal-Wallis | Dunn MRD | 57.80 | -26.01 | 3 | <0.0001 (****) | 0.003 (**) | 30 |
| S1A – NLGN3 | Axon count/cell | CTRL – NLGN3 | Y | 2-way ANOVA | Bonf | 24.00 | 7.79 | 2,240 | <0.0001 (****) | <0.0001 (****) | 15 |
| S1A – NLGN3 | Dendrite count/cell | CTRL – NLGN3 | Y | 2-way ANOVA | Bonf | 24.00 | 4.42 | 2,240 | <0.0001 (****) | <0.0001 (****) | 15 |
| S1B – NLGN3 | Axon length/cell | CTRL – NLGN3 | Y | 2-way ANOVA | Bonf | 21.76 | 10.05 | 2,240 | <0.0001 (****) | 0.005 (**) | 15 |
| S1B – NLGN3 | Axon length/cell | CTRL – NLGN3-R451C | Y | 2-way ANOVA | Bonf | 21.76 | 3.17 | 2,240 | <0.0001 (****) | <0.0001 (****) | 15 |
| S1C – NLGN4X | Axon count/cell | CTRL – NLGN4X-WT | Y | 2-way ANOVA | Bonf | 35.61 | 8.78 | 2,212 | <0.0001 (****) | <0.0001 (****) | 15 |
| S1C – NLGN4X | Dendrite count/cell | CTRL – NLGN4X-WT | Y | 2-way ANOVA | Bonf | 35.61 | 4.81 | 2,212 | <0.0001 (****) | <0.0001 (****) | 15 |
| S1D – NLGN4X | Axon length/cell | CTRL – NLGN4X-WT | Y | 2-way ANOVA | Bonf | 34.65 | 10.78 | 2,212 | <0.0001 (****) | <0.0001 (****) | 15 |
| S1D – NLGN4X | Dendrite length/cell | CTRL – NLGN4X-WT | Y | 2-way ANOVA | Bonf | 34.65 | 5.15 | 2,212 | <0.0001 (****) | <0.0001 (****) | 15 |

| | | | | | | | | | | | |
|---|---|---|---|---|---|---|---|---|---|---|---|
| S1E – NLGN3/4X | Axon count/cell | NLGN3-WT – NLGN4X-WT | Y | 2-way ANOVA | Bonf | 23.66 | 1.33 | 2,230 | <0.0001 (****) | 0.56 (n.s.) | 15 |
| S1E – NLGN3/4X | Dendrite count/cell | NLGN3-WT – NLGN4X-WT | Y | 2-way ANOVA | Bonf | 23.66 | 0.87 | 2,230 | <0.0001 (****) | >0.99 (n.s.) | 15 |
| S1F – NLGN3/4X | Axon length/cell | NLGN3-WT – NLGN4X-WT | Y | 2-way ANOVA | Bonf | 22.94 | 1.05 | 2,230 | <0.0001 (****) | 0.89 (n.s.) | 15 |
| S1F – NLGN3/4X | Dendrite length/cell | NLGN3-WT – NLGN4X-WT | Y | 2-way ANOVA | Bonf | 22.94 | 0.86 | 2,230 | <0.0001 (****) | >0.99 (n.s.) | 15 |
| 2B – NLGN4X | Neurite count/cell | CTRL – NLGN4X-WT | N | Kruskal-Wallis | Dunn MRD | 56.31 | -44.70 | 3 | <0.0001 (****) | <0.0001 (****) | 30 |
| 2C – NLGN4X | Neurite length/cell | CTRL – NLGN4X-WT | N | Kruskal-Wallis | Dunn MRD | 55.07 | -50.42 | 3 | <0.0001 (****) | <0.0001 (****) | 30 |
| 3A – NLGN3 | RNA fold-change | NPC – Neuron | N/A | t test | N/A | 8.822 | N/A | 4 | 0.0009 (***) | N/A | 3 |
| 3A – NLGN4X | RNA fold-change | NPC – Neuron | N/A | t test | N/A | 0.134 | N/A | 4 | 0.134 | N/A | 3 |
| 3B – NLGN3 | Protein expression | NPC – Neuron | N/A | t test | N/A | 3.25 | N/A | 4 | 0.031 (*) | N/A | 3 |
| 3B – NLGN4X | Protein expression | NPC – Neuron | N/A | t test | N/A | 3.35 | N/A | 4 | 0.029 (*) | N/A | 3 |
| S7A-B – NLGN3 | Count/cell, lamellipodia on protrusions | CTRL – NLGN3-WT | Y | 1-way ANOVA | Bonf | 2.58 | 3.39 | 4,126 | 0.041 (*) | 0.003 (**) | 15 |
| 4C-D – NLGN3 | Neurite count/cell | CTRL – NLGN3-WT | N | Kruskal-Wallis | Dunn MRD | 18.07 | -14.37 | 3 | 0.0001 (***) | 0.008 (**) | 15 |
| 4C-D – NLGN3 | Neurite length/cell | CTRL – NLGN3 | Y | 1-way ANOVA | Bonf | 15.97 | 4.41 | 2,42 | <0.0001 (****) | <0.0001 (****) | 15 |
| S7C – NLGN3 | 2$^{nd}$ neurite count/cell | CTRL – NLGN3 | Y | 2-way ANOVA | Bonf | 3.537 | 4.72 | 4,126 | 0.009 (**) | <0.0001 (****) | 15 |

| | | | | | | | | | | | |
|---|---|---|---|---|---|---|---|---|---|---|---|
| S7C – NLGN3 | 2nd neurite length/cell | CTRL – NLGN3 | Y | 2-way ANOVA | Bonf | 2.873 | 5.18 | 4,126 | 0.026 (*) | <0.0001 (****) | 15 |
| S7A-B – NLGN4X | Count/cell, concaves | CTRL – NLGN4X-D396 | Y | 1-way ANOVA | Bonf | 23.84 | 5.13 | 4,120 | <0.0001 (****) | <0.0001 (****) | 15 |
| S7A-B – NLGN4X | Count/cell, lamellipodia | CTRL – NLGN4X-D396 | Y | 1-way ANOVA | Bonf | 23.84 | 2.52 | 4,120 | <0.0001 (****) | 0.039 (*) | 15 |
| S7A-B – NLGN4X | Count/cell, lamellipodia on protrusions | CTRL – NLGN4X-D396 | Y | 1-way ANOVA | Bonf | 23.84 | 3.80 | 4,120 | <0.0001 (****) | 0.0007 (***) | 15 |
| 4E-F – NLGN4X | Neurite count/cell | CTRL-NLGN4X-WT | Y | 1-way ANOVA | Bonf | 11.62 | 2.60 | 2,42 | <0.0001 (****) | 0.039 (*) | 15 |
| 4E-F – NLGN4X | Neurite length/cell | CTRL-NLGN4X-WT | N | Kruskal-Wallis | Dunn MRD | 26.95 | -13.53 | 3 | <.00001 (****) | 0.014 (*) | 15 |
| S7D – NLGN4X | 2nd neurite count/cell | CTRL – NLGN4X-WT | Y | 2-way ANOVA | Bonf | 14.76 | 3.09 | 4,126 | <0.0001 (****) | 0.008 (**) | 15 |
| S7D – NLGN4X | 2nd neurite length/cell | CTRL – NLGN4X-WT | Y | 2-way ANOVA | Bonf | 4.267 | 5.74 | 4,126 | 0.0028 (**) | <0.0001 (****) | 15 |
| 5A-B – NLGN3 | Growth cone area | CTRL – NLGN3 | Y | 1-way ANOVA | Bonf | 6.434 | 2.86 | 2,35 | 0.004 (**) | 0.021 (*) | 15 |
| 5A-B – NLGN3 | Filament no. | CTRL – NLGN3-WT | Y | 1-way ANOVA | Bonf | 15.57 | 4.43 | 2,44 | <0.0001 (****) | 0.0002 (***) | 15 |
| 5A-B – NLGN3 | Filament length | CTRL – NLGN3-WT | Y | 1-way ANOVA | Bonf | 4.83 | 2.73 | 2,39 | 0.0134 (*) | 0.028 (*) | 15 |
| 5A-B – NLGN3 | Bundle width | CTRL – NLGN3-WT | Y | 1-way ANOVA | Bonf | 33.47 | 6.10 | 2,44 | <0.0001 (****) | <0.0001 (****) | 15 |
| 5A-B – NLGN3 | Filament distance (normalized) | CTRL – NLGN3-WT | N | Kruskal-Wallis | Dunn MRD | 22.07 | 17.41 | 3 | <0.0001 (****) | 0.001 (**) | |

| | | | | | | | | | | | |
|---|---|---|---|---|---|---|---|---|---|---|---|
| 5A-B – NLGN3 | Anisotropy | CTRL – NLGN3-WT | Y | 1-way ANOVA | Bonf | 6.621 | 3.21 | 2,43 | 0.0031 (***) | 0.0075 (***) | 15 |
| 5C-D – NLGN4X | Growth cone area | CTRL – NLGN4X-WT | Y | 1-way ANOVA | Bonf | 15.54 | 4.22 | 2,45 | <0.0001 (****) | 0.0003 (***) | 15 |
| 5C-D – NLGN4X | Filament no. | CTRL – NLGN4X-WT | Y | 1-way ANOVA | Bonf | 12.38 | 3.51 | 2,46 | <0.0001 (****) | 0.003 (***) | 15 |
| 5C-D – NLGN4X | Filament length | CTRL – NLGN4X-WT | N | Kruskal-Wallis | Dunn MRD | 16.06 | -13.47 | 3 | 0.0003 (***) | 0.028 (*) | 15 |
| 5C-D – NLGN4X | Bundle width | CTRL – NLGN4X-WT | Y | 1-way ANOVA | Bonf | 13.02 | 3.55 | 2,46 | <0.0001 (****) | 0.003 (**) | 15 |
| 5C-D – NLGN4X | Filament distance (normalized) | CTRL – NLGN4X-WT | N | Kruskal-Wallis | Dunn MRD | 27.28 | 12.45 | 3 | <0.0001 (****) | 0.043 (*) | 15 |
| 5C-D – NLGN4X | Filament distance (normalized) | CTRL – NLGN4X-D396 | N | Kruskal-Wallis | Dunn MRD | 27.28 | -12.04 | 3 | <0.0001 | 0.041 (*) | 15 |
| 5C-D – NLGN4X | Anisotropy | CTRL – NLGN4X – NLGN4X-D396 | N | Kruskal-Wallis | N/A | 2.59 | N/A | 3 | 0.27 (n.s.) | N/A | 15 |
| 6A – NLGN3 | PAK1 Phosphorylation | CTRL – NLGN3-WT | Y | 1-way ANOVA | Bonf | 13.97 | 5.19 | 2,6 | 0.006 (**) | 0.006 (**) | 3 |
| 6A – NLGN3 | Cofilin Phosphorylation | CTRL – NLGN3-WT | Y | 1-way ANOVA | Bonf | 160.20 | 17.70 | 2,6 | <0.0001 (****) | <0.0001 (****) | 3 |
| 6B – NLGN4X | PAK1 Phosphorylation | CTRL – NLGN4X-WT | Y | 1-way ANOVA | Bonf | 26.15 | 4.13 | 2,6 | 0.001 (***) | 0.02 (*) | 3 |
| 6B – NLGN4X | Cofilin Phosphorylation | CTRL – NLGN4X-WT | Y | 1-way ANOVA | Bonf | 35.95 | 6.73 | 2,6 | 0.0005 (***) | 0.002 (**) | 3 |
| 6E – Shank3 | PAK1 Phosphorylation | CTRL – Shank3 | Y | 1-way ANOVA | Bonf | 28.09 | 5.22 | 5,12 | <0.0001 (****) | 0.0286 (*) | 3 |

| | | | | | | | | | | | |
|---|---|---|---|---|---|---|---|---|---|---|---|
| 6E – Shank3 | PAK1 Phosphorylation | CTRL – NLGN3-WT | Y | 1-way ANOVA | Bonf | 28.09 | 7.77 | 5,12 | <0.0001 (****) | 0.0015 (**) | 3 |
| 6E – Shank3 | PAK1 Phosphorylation | CTRL – NLGN4X-WT | Y | 1-way ANOVA | Bonf | 28.09 | 7.00 | 5,12 | <0.0001 (****) | 0.0035 (**) | 3 |
| 6E – Shank3 | PAK1 Phosphorylation | CTRL – Sh3+NL3-WT | Y | 1-way ANOVA | Bonf | 28.09 | 10.46 | 5,12 | <0.0001 (****) | <0.0001 (****) | 3 |
| 6E – Shank3 | PAK1 Phosphorylation | CTRL – Sh3+NL4X-WT | Y | 1-way ANOVA | Bonf | 28.09 | 15.88 | 5,12 | <0.0001 (****) | <0.0001 (****) | 3 |
| 6E – Shank3 | PAK1 Phosphorylation | Sh3 – Sh3+NL3-WT | Y | 1-way ANOVA | Bonf | 28.09 | 5.24 | 5,12 | <0.0001 (****) | 0.028 (*) | 3 |
| 6E – Shank3 | PAK1 Phosphorylation | Sh3 – Sh3+NL4X-WT | Y | 1-way ANOVA | Bonf | 28.09 | 10.66 | 5,12 | <0.0001 (****) | <0.0001 (****) | 3 |
| 6E – Shank3 | PAK1 Phosphorylation | Sh3+NL3 – Sh3+NL4X | Y | 1-way ANOVA | Bonf | 28.09 | 5.43 | 5,12 | <0.0001 (****) | 0.022 (*) | 3 |
| 7A-B – NLGN3 | Growth cone area | CTRL-DMSO – NLGN3-FRAX | N | 2-way ANOVA | Bonf | 35.97 | 0.81 | 1,85 | <0.0001 (****) | >0.99 (n.s.) | 25 |
| 7A-B – NLGN3 | Filament no. | CTRL-DMSO – NLGN3-FRAX | N | 2-way ANOVA | Bonf | 51.23 | 1.66 | 1,91 | <0.0001 (****) | >0.99 (n.s.) | 25 |
| 7A-B – NLGN3 | Filament distance (normalized) | CTRL-DMSO – NLGN3-FRAX | N | 2-way ANOVA | Bonf | 28.30 | 0.16 | 1,91 | <0.0001 (****) | >0.99 (n.s.) | 25 |
| 7A-C – NLGN4X | Growth cone area | CTRL-DMSO – NLGN4X-FRAX | N | 2-way ANOVA | Bonf | 43.61 | 1.64 | 1,74 | <0.0001 (****) | >0.99 (n.s.) | 25 |

| | | | | | | | | | | | |
|---|---|---|---|---|---|---|---|---|---|---|---|
| 7A-C – NLGN4X | Filament no. | CTRL-DMSO – NLGN4X-FRAX | N | 2-way ANOVA | Bonf | 65.66 | 3.73 | 1,81 | <0.0001 (****) | >0.99 (n.s.) | 25 |
| 7A-C – NLGN4X | Filament distance (normalized) | CTRL-DMSO – NLGN4X-FRAX | N | 2-way ANOVA | Bonf | 21.59 | 0.37 | 1,81 | <0.0001 (****) | >0.99 (n.s.) | 25 |
| 8A – NLGN3 | Phospho-PAK1 clusters | CTRL – NLGN3-WT | Y | t test | N/A | 2.07 | N/A | 43 | 0.04 (*) | N/A | 25 |
| 8A – NLGN4X | Phospho-PAK1 clusters | CTRL – NLGN4X-WT | Y | t test | N/A | 3.52 | N/A | 41 | 0.001 (**) | N/A | 23 |
| 8B – NLGN3 | Cluster no. | DMSO – FRAX | Y | t test | N/A | 5.16 | N/A | 47 | <0.0001 (****) | N/A | 25 |
| 8B – NLGN3 | Cluster area | DMSO – FRAX | N | Mann-Whitney | N/A | 100 | N/A | N/A | <0.0001 (****) | N/A | 25 |
| 8B – NLGN3 | Cluster intensity | DMSO – FRAX | N | Mann-Whitney | N/A | 117 | N/A | N/A | 0.0096 (**) | N/A | 25 |
| S9A – NLGN3 | Cluster no. (GC area norm’d) | DMSO – FRAX | N | Mann-Whitney | N/A | 127 | N/A | N/A | 0.0021 (**) | N/A | 25 |
| 8C – NLGN4X | Cluster no. | DMSO – FRAX | Y | t test | N/A | 4.76 | N/A | 37 | <0.0001 (****) | N/A | 25 |
| 8C – NLGN4X | Cluster area | DMSO – FRAX | N | Mann-Whitney | N/A | 41 | N/A | N/A | <0.0001 (****) | N/A | 25 |
| 8C – NLGN4X | Cluster intensity | DMSO – FRAX | N | Mann-Whitney | N/A | 87 | N/A | N/A | 0.015 (*) | N/A | 25 |
| S9B – NLGN4X | Cluster no. (GC area norm’d) | DMSO – FRAX | N | Mann-Whitney | N/A | 79 | N/A | N/A | 0.0022 (**) | N/A | 25 |
| 9A – NLGN3 | Neurite number | CTRL-DMSO – NLGN3-FRAX | N | 2-way ANOVA | Bonf | 27.77 | 0.47 | 1,85 | <0.0001 (****) | >0.99 (n.s.) | 25 |

| | | | | | | | | | | | |
|---|---|---|---|---|---|---|---|---|---|---|---|
| 9A – NLGN3 | Neurite length | CTRL-DMSO – NLGN3-FRAX | N | 2-way ANOVA | Bonf | 98.78 | 2.46 | 1,84 | <0.0001 (****) | 0.10 (n.s.) | 25 |
| 9B – NLGN4X | Neurite number | CTRL-DMSO – NLGN4X-FRAX | N | 2-way ANOVA | Bonf | 55.36 | 0.35 | 1,78 | <0.0001 (****) | >0.99 (n.s.) | 25 |
| 9B – NLGN4X | Neurite length | CTRL-DMSO – NLGN4X-FRAX | N | 2-way ANOVA | Bonf | 108.70 | 1.59 | 1,77 | <0.0001 (****) | >0.99 (n.s.) | 25 |

**Table S3 –** A table detailing all statistics used in this study.

**Key**: NLGN – Neuroligin, ND? – Normally distributed?, MC – Multiple Corrections, df – degrees of freedom, sig – significance, Adj p value – Adjusted p value, n – sample size, NPC – Neural progenitor cell, Y – Yes, N – No, Bonf – Bonferroni post-hoc correction, Dunn MRD – Dunn's Mean Rank Difference, n.s. – Not significant, PAK1 – p21-activated kinase.

Gatford et al. Table S3

| Figure | Variable | Condition | Mean | ±SEM | Unit |
|---|---|---|---|---|---|
| 1B – NLGN3 | Neurite number | CTRL | 4.5 | 0.21 | A.U. |
| 1B – NLGN3 | Neurite number | NLGN3-WT | 11.07 | 0.78 | A.U. |
| 1B – NLGN3 | Neurite number | NLGN3-R451C | 6.64 | 0.53 | A.U. |
| 1C – NLGN3 | Neurite length | CTRL | 162.1 | 9.45 | µm |
| 1C – NLGN3 | Neurite length | NLGN3-WT | 487.4 | 32.73 | µm |
| 1C – NLGN3 | Neurite length | NLGN3-R451C | 280.5 | 26.14 | µm |
| 2B – NLGN4X | Neurite number | CTRL | 4.5 | 0.21 | A.U. |
| 2B – NLGN4X | Neurite number | NLGN4X-WT | 9.92 | 0.59 | A.U. |
| 2B – NLGN4X | Neurite number | NLGN4X-D396 | 4.15 | 0.28 | A.U. |
| 2C – NLGN4X | Neurite length | CTRL | 162.1 | 9.45 | µm |
| 2C – NLGN4X | Neurite length | NLGN4X-WT | 489.1 | 32.68 | µm |
| 2C – NLGN4X | Neurite length | NLGN4X-D396 | 202.2 | 19.29 | µm |
| 3B – NLGN3 | Protein expression | NPC | 0.19 | 0.04 | A.U. |
| 3B – NLGN3 | Protein expression | Neuron | 0.35 | 0.03 | A.U. |
| 3B – NLGN4X | Protein expression | NPC | 1.54 | 1.07 | A.U. |
| 3B – NLGN4X | Protein expression | Neuron | 5.28 | 0.32 | A.U. |
| 4C-D – NLGN3 | Neurite count/cell | CTRL | 10.67 | 1.01 | A.U. |
| 4C-D – NLGN3 | Neurite count/cell | NLGN3-WT | 17.80 | 1.67 | A.U. |
| 4C-D – NLGN3 | Neurite count/cell | NLGN3-R451C | 8.60 | 1.17 | A.U. |
| 4C-D – NLGN3 | Neurite length/cell | CTRL | 203.20 | 16.13 | µm |
| 4C-D – NLGN3 | Neurite length/cell | NLGN3-WT | 356.40 | 31.79 | µm |

| | | | | | |
|---|---|---|---|---|---|
| 4C-D – NLGN3 | Neurite length/cell | NLGN3-R451C | 173.10 | 23.36 | μm |
| 4E-F – NLGN4X | Neurite count/cell | CTRL | 10.67 | 1.01 | A.U. |
| 4E-F – NLGN4X | Neurite count/cell | NLGN4X-WT | 15.67 | 1.90 | A.U. |
| 4E-F – NLGN4X | Neurite count/cell | NLGN4X-D396 | 6.40 | 0.96 | A.U. |
| 4E-F – NLGN4X | Neurite length/cell | CTRL | 203.20 | 16.13 | μm |
| 4E-F – NLGN4X | Neurite length/cell | NLGN4X-WT | 332.40 | 25.36 | μm |
| 4E-F – NLGN4X | Neurite length/cell | NLGN4X-D396 | 114.00 | 17.40 | μm |
| 5A-B – NLGN3 | Growth cone area | CTRL | 253.20 | 28.67 | $\mu m^2$ |
| 5A-B – NLGN3 | Growth cone area | NLGN3-WT | 404.70 | 51.90 | $\mu m^2$ |
| 5A-B – NLGN3 | Growth cone area | NLGN3-R451C | 218.10 | 33.31 | $\mu m^2$ |
| 5A-B – NLGN3 | Filament no. | CTRL | 9.80 | 1.22 | A.U. |
| 5A-B – NLGN3 | Filament no. | NLGN3-WT | 17.80 | 1.78 | A.U. |
| 5A-B – NLGN3 | Filament no. | NLGN3-R451C | 8.71 | 0.60 | A.U. |
| 5A-B – NLGN3 | Filament length | CTRL | 3.19 | 0.33 | μm |
| 5A-B – NLGN3 | Filament length | NLGN3-WT | 4.06 | 0.36 | μm |
| 5A-B – NLGN3 | Filament length | NLGN3-R451C | 3.21 | 0.18 | μm |
| 5A-B – NLGN3 | Bundle width | CTRL | 0.36 | 0.04 | μm |
| 5A-B – NLGN3 | Bundle width | NLGN3-WT | 0.84 | 0.09 | μm |
| 5A-B – NLGN3 | Bundle width | NLGN3-R451C | 0.25 | 0.03 | μm |
| 5A-B – NLGN3 | Filament distance (normalized) | CTRL | 0.16 | 0.02 | μm |
| 5A-B – NLGN3 | Filament distance (normalized) | NLGN3-WT | 0.06 | 0.01 | μm |

| | | | | | |
|---|---|---|---|---|---|
| 5A-B – NLGN3 | Filament distance (normalized) | NLGN3-R451C | 0.22 | 0.03 | µm |
| 5A-B – NLGN3 | Anisotropy | CTRL | 0.16 | 0.02 | A.U. |
| 5A-B – NLGN3 | Anisotropy | NLGN3-WT | 0.07 | 0.02 | A.U. |
| 5A-B – NLGN3 | Anisotropy | NLGN3-R451C | 0.16 | 0.02 | A.U. |
| 5C-D – NLGN4X | Growth cone area | CTRL | 253.20 | 28.67 | $\mu m^2$ |
| 5C-D – NLGN4X | Growth cone area | NLGN4X-WT | 598.90 | 95.19 | $\mu m^2$ |
| 5C-D – NLGN4X | Growth cone area | NLGN4X-D396 | 182.50 | 24.85 | $\mu m^2$ |
| 5C-D – NLGN4X | Filament no. | CTRL | 9.80 | 1.22 | A.U. |
| 5C-D – NLGN4X | Filament no. | NLGN4X-WT | 18.20 | 2.74 | A.U. |
| 5C-D – NLGN4X | Filament no. | NLGN4X-D396 | 7.21 | 0.53 | A.U. |
| 5C-D – NLGN4X | Filament length | CTRL | 3.20 | 0.09 | µm |
| 5C-D – NLGN4X | Filament length | NLGN4X-WT | 4.69 | 0.62 | µm |
| 5C-D – NLGN4X | Filament length | NLGN4X-D396 | 3.14 | 0.24 | µm |
| 5C-D – NLGN4X | Bundle width | CTRL | 0.36 | 0.04 | µm |
| 5C-D – NLGN4X | Bundle width | NLGN4X-WT | 0.76 | 0.14 | µm |
| 5C-D – NLGN4X | Bundle width | NLGN4X-D396 | 0.23 | 0.02 | µm |
| 5C-D – NLGN4X | Filament distance (normalized) | CTRL | 0.16 | 0.02 | µm |
| 5C-D – NLGN4X | Filament distance (normalized) | NLGN4X-WT | 0.08 | 0.01 | µm |
| 5C-D – NLGN4X | Filament distance (normalized) | NLGN4X-D396 | 0.30 | 0.03 | µm |
| 5C-D – NLGN4X | Anisotropy | CTRL | 0.16 | 0.03 | A.U. |

| | | | | | |
|---|---|---|---|---|---|
| 5C-D – NLGN4X | Anisotropy | NLGN4X-WT | 0.14 | 0.03 | A.U. |
| 5C-D – NLGN4X | Anisotropy | NLGN4X-D396 | 0.13 | 0.03 | A.U. |
| 6A – NLGN3 | PAK1 Phosphorylation | CTRL | 1.00 | 0.37 | A.U. |
| 6A – NLGN3 | PAK1 Phosphorylation | NLGN3-WT | 3.10 | 0.32 | A.U. |
| 6A – NLGN3 | PAK1 Phosphorylation | NLGN3-R451C | 1.70 | 0.05 | A.U. |
| 6A – NLGN3 | Cofilin Phosphorylation | CTRL | 1.00 | 0.37 | A.U. |
| 6A – NLGN3 | Cofilin Phosphorylation | NLGN3-WT | 1.70 | 0.01 | A.U. |
| 6A – NLGN3 | Cofilin Phosphorylation | NLGN3-R451C | 1.26 | 0.04 | A.U. |
| 6B – NLGN4X | PAK1 Phosphorylation | CTRL | 1.00 | 0.05 | A.U. |
| 6B – NLGN4X | PAK1 Phosphorylation | NLGN4X-WT | 1.56 | 0.11 | A.U. |
| 6B – NLGN4X | PAK1 Phosphorylation | NLGN4X-D396 | 0.58 | 0.11 | A.U. |
| 6B – NLGN4X | Cofilin Phosphorylation | CTRL | 1.00 | 0.09 | A.U. |
| 6B – NLGN4X | Cofilin Phosphorylation | NLGN4X-WT | 1.61 | 0.05 | A.U. |
| 6B – NLGN4X | Cofilin Phosphorylation | NLGN4X-D396 | 0.90 | 0.05 | A.U. |
| 6E – Shank3 | PAK1 Phosphorylation | CTRL | 1.00 | 0.00 | A.U. |
| 6E – Shank3 | PAK1 Phosphorylation | Shank3 | 2.79 | 0.06 | A.U. |
| 6E – Shank3 | PAK1 Phosphorylation | NLGN3-WT | 3.67 | 0.09 | A.U. |
| 6E – Shank3 | PAK1 Phosphorylation | NLGN4X-WT | 3.40 | 0.15 | A.U. |
| 6E – Shank3 | PAK1 Phosphorylation | Shank3+NLGN3-WT | 4.59 | 0.18 | A.U. |
| 6E – Shank3 | PAK1 Phosphorylation | Shank3+NLGN4X-WT | 6.45 | 1.43 | A.U. |

| | | | | | |
|---|---|---|---|---|---|
| 7A-B – NLGN3 | Growth cone area | CTRL-DMSO | 172.30 | 11.02 | $\mu m^2$ |
| 7A-B – NLGN3 | Growth cone area | CTRL-FRAX | 88.02 | 9.27 | $\mu m^2$ |
| 7A-B – NLGN3 | Growth cone area | NLGN3-DMSO | 468.83 | 48.98 | $\mu m^2$ |
| 7A-B – NLGN3 | Growth cone area | NLGN3-FRAX | 205.76 | 19.01 | $\mu m^2$ |
| 7A-B – NLGN3 | Filament no. | CTRL-DMSO | 10.68 | 0.71 | A.U. |
| 7A-B – NLGN3 | Filament no. | CTRL-FRAX | 3.09 | 0.33 | A.U. |
| 7A-B – NLGN3 | Filament no. | NLGN3-DMSO | 23.40 | 1.48 | A.U. |
| 7A-B – NLGN3 | Filament no. | NLGN3-FRAX | 11.95 | 0.79 | A.U. |
| 7A-B – NLGN3 | Filament distance (normalized) | CTRL-DMSO | 0.10 | 0.01 | µm |
| 7A-B – NLGN3 | Filament distance (normalized) | CTRL-FRAX | 0.44 | 0.06 | µm |
| 7A-B – NLGN3 | Filament distance (normalized) | NLGN3-DMSO | 0.04 | 0.004 | µm |
| 7A-B – NLGN3 | Filament distance (normalized) | NLGN3-FRAX | 0.08 | 0.008 | µm |
| 7A-C – NLGN4X | Growth cone area | CTRL-DMSO | 172.30 | 11.02 | $\mu m^2$ |
| 7A-C – NLGN4X | Growth cone area | CTRL-FRAX | 88.02 | 9.27 | $\mu m^2$ |
| 7A-C – NLGN4X | Growth cone area | NLGN4X-DMSO | 432.00 | 32.09 | $\mu m^2$ |
| 7A-C – NLGN4X | Growth cone area | NLGN4X-FRAX | 214.70 | 13.40 | $\mu m^2$ |
| 7A-C – NLGN4X | Filament no. | CTRL-DMSO | 10.68 | 0.71 | A.U. |
| 7A-C – NLGN4X | Filament no. | CTRL-FRAX | 3.09 | 0.33 | A.U. |
| 7A-C – NLGN4X | Filament no. | NLGN4X-DMSO | 28.18 | 1.81 | A.U. |
| 7A-C – NLGN4X | Filament no. | NLGN4X-FRAX | 16.18 | 0.85 | A.U. |

| | | | | | |
|---|---|---|---|---|---|
| 7A-C – NLGN4X | Filament distance (normalized) | CTRL-DMSO | 0.10 | 0.01 | µm |
| 7A-C – NLGN4X | Filament distance (normalized) | CTRL-FRAX | 0.44 | 0.06 | µm |
| 7A-C – NLGN4X | Filament distance (normalized) | NLGN4X-DMSO | 0.03 | 0.002 | µm |
| 7A-C – NLGN4X | Filament distance (normalized) | NLGN4X-FRAX | 0.06 | 0.006 | µm |
| 8A – NLGN3 | Phospho-PAK1 cluster intensity | CTRL | 11.04 | 2.22 | A.U. |
| 8A – NLGN3 | Phospho-PAK1 cluster intensity | NLGN3-WT | 27.62 | 6.92 | A.U. |
| 8A – NLGN4X | Phospho-PAK1 cluster intensity | CTRL | 11.04 | 2.22 | A.U. |
| 8A – NLGN4X | Phospho-PAK1 cluster intensity | NLGN4X-WT | 35.39 | 6.14 | A.U. |
| 8B – NLGN3 | Cluster no. | NLGN3-DMSO | 863.80 | 127.50 | A.U. |
| 8B – NLGN3 | Cluster no. | NLGN3-FRAX | 158.70 | 15.29 | A.U. |
| 8B – NLGN3 | Cluster area | NLGN3-DMSO | 0.17 | 0.04 | $\mu m^2$ |
| 8B – NLGN3 | Cluster area | NLGN3-FRAX | 0.05 | 0.008 | $\mu m^2$ |
| 8B – NLGN3 | Cluster intensity | NLGN3-DMSO | 11.46 | 3.53 | A.U. |
| 8B – NLGN3 | Cluster intensity | NLGN3-FRAX | 4.38 | 1.09 | A.U. |
| 8C – NLGN4X | Cluster no. | NLGN4X-DMSO | 713.70 | 83.84 | A.U. |
| 8C – NLGN4X | Cluster no. | NLGN4X-FRAX | 212.80 | 35.68 | A.U. |
| 8C – NLGN4X | Cluster area | NLGN4X-DMSO | 0.21 | 0.05 | $\mu m^2$ |
| 8C – NLGN4X | Cluster area | NLGN4X-FRAX | 0.06 | 0.01 | $\mu m^2$ |
| 8C – NLGN4X | Cluster intensity | NLGN4X-DMSO | 10.44 | 2.32 | A.U. |
| 8C – NLGN4X | Cluster intensity | NLGN4X-FRAX | 3.81 | 0.86 | A.U. |

| | | | | | |
|---|---|---|---|---|---|
| 9A – NLGN3 | Neurite number | CTRL-DMSO | 7.05 | 0.43 | A.U. |
| 9A – NLGN3 | Neurite number | CTRL-FRAX | 3.14 | 0.24 | A.U. |
| 9A – NLGN3 | Neurite number | NLGN3-DMSO | 14.00 | 1.16 | A.U. |
| 9A – NLGN3 | Neurite number | NLGN3-FRAX | 6.26 | 0.37 | A.U. |
| 9A – NLGN3 | Neurite length | CTRL-DMSO | 181.00 | 8.32 | µm |
| 9A – NLGN3 | Neurite length | CTRL-FRAX | 59.89 | 6.00 | µm |
| 9A – NLGN3 | Neurite length | NLGN3-DMSO | 302.20 | 22.45 | µm |
| 9A – NLGN3 | Neurite length | NLGN3-FRAX | 130.40 | 8.87 | µm |
| 9B – NLGN4X | Neurite number | CTRL-DMSO | 7.05 | 0.43 | A.U. |
| 9B – NLGN4X | Neurite number | CTRL-FRAX | 3.14 | 0.24 | A.U. |
| 9B – NLGN4X | Neurite number | NLGN4X-DMSO | 13.61 | 1.03 | A.U. |
| 9B – NLGN4X | Neurite number | NLGN4X-FRAX | 7.31 | 0.58 | A.U. |
| 9B – NLGN4X | Neurite length | CTRL-DMSO | 181.00 | 8.32 | µm |
| 9B – NLGN4X | Neurite length | CTRL-FRAX | 59.89 | 6.00 | µm |
| 9B – NLGN4X | Neurite length | NLGN4X-DMSO | 276.40 | 15.08 | µm |
| 9B – NLGN4X | Neurite length | NLGN4X-FRAX | 153.30 | 13.44 | µm |

**Table S4 –** A table detailing all raw data presented in the main figures of this study.

**Key**: NLGN – Neuroligin, SEM – Standard Error of the Mean, NPC – Neural progenitor cell, A.U. – Arbitrary Unit, CTRL – Control, WT – Wild Type, PAK1 – p21-activated kinase, DMSO – Dimethyl Sulfoxide, FRAX – FRAX486.

| Figure | Variable | Condition | Mean | ±SEM | Unit |
|---|---|---|---|---|---|
| S1A – NLGN3 | Axon count/cell | CTRL | 2.18 | 0.16 | A.U. |
| S1A – NLGN3 | Axon count/cell | NLGN3-WT | 6.47 | 0.56 | A.U. |
| S1A – NLGN3 | Axon count/cell | NLGN3-R451C | 3.43 | 0.42 | A.U. |
| S1A – NLGN3 | Dendrite count/cell | CTRL | 2.32 | 0.19 | A.U. |
| S1A – NLGN3 | Dendrite count/cell | NLGN3-WT | 4.74 | 0.47 | A.U. |
| S1A – NLGN3 | Dendrite count/cell | NLGN3-R451C | 3.19 | 0.25 | A.U. |
| S1B – NLGN3 | Axon length/cell | CTRL | 104.70 | 8.65 | µm |
| S1B – NLGN3 | Axon length/cell | NLGN3-WT | 354.92 | 29.62 | µm |
| S1B – NLGN3 | Axon length/cell | NLGN3-R451C | 184.19 | 21.71 | µm |
| S1B – NLGN3 | Dendrite length/cell | CTRL | 56.56 | 5.39 | µm |
| S1B – NLGN3 | Dendrite length/cell | NLGN3-WT | 139.92 | 13.46 | µm |
| S1B – NLGN3 | Dendrite length/cell | NLGN3-R451C | 95.90 | 8.80 | µm |
| S1C – NLGN4X | Axon count/cell | CTRL | 2.18 | 0.16 | A.U. |
| S1C – NLGN4X | Axon count/cell | NLGN4X-WT | 5.70 | 0.44 | A.U. |
| S1C – NLGN4X | Axon count/cell | NLGN4X-D396 | 1.47 | 0.14 | A.U. |
| S1C – NLGN4X | Dendrite count/cell | CTRL | 2.32 | 0.19 | A.U. |
| S1C – NLGN4X | Dendrite count/cell | NLGN4X-WT | 4.24 | 0.38 | A.U. |
| S1C – NLGN4X | Dendrite count/cell | NLGN4X-D396 | 2.65 | 0.26 | A.U. |
| S1D – NLGN4X | Axon length/cell | CTRL | 104.70 | 8.65 | µm |

| | | | | | |
|---|---|---|---|---|---|
| S1D – NLGN4X | Axon length/cell | NLGN4X-WT | 327.11 | 23.51 | µm |
| S1D – NLGN4X | Axon length/cell | NLGN4X-D396 | 99.85 | 13.93 | µm |
| S1D – NLGN4X | Dendrite length/cell | CTRL | 56.56 | 5.39 | µm |
| S1D – NLGN4X | Dendrite length/cell | NLGN4X-WT | 162.65 | 17.66 | µm |
| S1D – NLGN4X | Dendrite length/cell | NLGN4X-D396 | 101.82 | 12.48 | µm |
| S7A-B – NLGN3 | Count/cell, lamellipodia on protrusions | CTRL | 4.33 | 0.47 | A.U. |
| S7A-B – NLGN3 | Count/cell, lamellipodia on protrusions | NLGN3-WT | 8.73 | 2.21 | A.U. |
| S7A-B – NLGN3 | Count/cell, lamellipodia on protrusions | NLGN3-R451C | 6.47 | 0.76 | A.U. |
| S7C – NLGN3 | $2^{nd}$ neurite count/cell | CTRL | 4.07 | 0.60 | A.U. |
| S7C – NLGN3 | $2^{nd}$ neurite count/cell | NLGN3-WT | 8.20 | 0.97 | A.U. |
| S7C – NLGN3 | $2^{nd}$ neurite count/cell | NLGN3-R451C | 3.20 | 0.72 | A.U. |
| S7C – NLGN3 | $2^{nd}$ neurite length/cell | CTRL | 58.73 | 8.61 | µm |
| S7C – NLGN3 | $2^{nd}$ neurite length/cell | NLGN3-WT | 152.14 | 19.78 | µm |
| S7C – NLGN3 | $2^{nd}$ neurite length/cell | NLGN3-R451C | 51.99 | 13.27 | µm |
| S7A-B – NLGN4X | Count/cell, concaves | CTRL | 4.87 | 0.62 | A.U. |
| S7A-B – NLGN4X | Count/cell, concaves | NLGN4X-D396 | 1.31 | 0.29 | A.U. |
| S7A-B – NLGN4X | Count/cell, lamellipodia | CTRL | 2.13 | 0.32 | A.U. |
| S7A-B – NLGN4X | Count/cell, lamellipodia | NLGN4X-D396 | 0.39 | 0.14 | A.U. |
| S7A-B – NLGN4X | Count/cell, lamellipodia on protrusions | CTRL | 4.33 | 0.47 | A.U. |
| S7A-B – NLGN4X | Count/cell, lamellipodia on protrusions | NLGN4X-D396 | 1.69 | 0.44 | A.U. |
| S7D – NLGN4X | $2^{nd}$ neurite count/cell | CTRL | 4.07 | 0.60 | A.U. |
| S7D – NLGN4X | $2^{nd}$ neurite count/cell | NLGN4X-WT | 8.20 | 0.97 | A.U. |
| S7D – NLGN4X | $2^{nd}$ neurite count/cell | NLGN4X-D396 | 3.20 | 0.72 | A.U. |

| | | | | | |
|---|---|---|---|---|---|
| S7D – NLGN4X | 2nd neurite length/cell | CTRL | 58.73 | 8.61 | µm |
| S7D – NLGN4X | 2nd neurite length/cell | NLGN4X-WT | 152.14 | 19.78 | µm |
| S7D – NLGN4X | 2nd neurite length/cell | NLGN4X-D396 | 51.99 | 13.27 | µm |
| S9A – NLGN3 | Cluster no. (GC area norm'd) | DMSO | 1.91 | 0.24 | A.U. |
| S9A – NLGN3 | Cluster no. (GC area norm'd) | FRAX | 0.91 | 0.13 | A.U. |
| S9B – NLGN4X | Cluster no. (GC area norm'd) | DMSO | 1.66 | 0.17 | A.U. |
| S9B – NLGN4X | Cluster no. (GC area norm'd) | FRAX | 0.95 | 0.13 | A.U. |

**Table S5 –** A table detailing all raw data presented in the supplemental figures of this study.

**Key**: NLGN – Neuroligin, SEM – Standard Error of the Mean, NPC – Neural progenitor cell, A.U. – Arbitrary Unit, CTRL – Control, WT – Wild Type, PAK1 – p21-activated kinase, DMSO – Dimethyl Sulfoxide, FRAX – FRAX486.

# References

1 Anderson, G.W., Deans, P.J., Taylor, R.D., Raval, P., Chen, D., Lowder, H., Murkerji, S., Andreae, L.C., Williams, B.P. and Srivastava, D.P. (2015) Characterisation of neurons derived from a cortical human neural stem cell line CTX0E16. *Stem Cell Research Therapy*, **6**, 149.

2 Pollock, K., Stroemer, P., Patel, S., Stevanato, L., Hope, A., Miljan, E., Dong, Z., Hodges, H., Price, J. and Sinden, J.D. (2006) A conditionally immortal clonal stem cell line from human cortical neuroepithelium for the treatment of ischemic stroke. *Experimental neurology*, **199**, 143-155.

3 Hayashi-Takagi, A., Araki, Y., Nakamura, M., Vollrath, B., Duron, S.G., Yan, Z., Kasai, H., Huganir, R.L., Campbell, D.A. and Sawa, A. (2014) PAKs inhibitors ameliorate schizophrenia-associated dendritic spine deterioration in vitro and in vivo during late adolescence. *Proceedings of the National Academy of Sciences*, **111**, 6461-6466.

4 Dolan, B.M., Duron, S.G., Campbell, D.A., Vollrath, B., Rao, B.S.S., Ko, H.-Y., Lin, G.G., Govindarajan, A., Choi, S.-Y. and Tonegawa, S. (2013) Rescue of fragile X syndrome phenotypes in Fmr1 KO mice by the small-molecule PAK inhibitor FRAX486. *Proceedings of the National Academy of Sciences of the United States of America*, **110**, 5671-5676.

5 Miller, J.A., Ding, S.-L., Sunkin, S.M., Smith, K.A., Ng, L., Szafer, A., Ebbert, A., Riley, Z.L., Royall, J.J., Aiona, K. *et al.* (2014) Transcriptional landscape of the prenatal human brain. *Nature*, **508**, 199-206.

6 Meijering, E., Jacob, M., Sarria, J.C., Steiner, P., Hirling, H. and Unser, M. (2004) Design and validation of a tool for neurite tracing and analysis in fluorescence microscopy images. *Cytometry Part A*, **58**, 167-176.

7 Steger, C. (1998) An unbiased detector of curvilinear structures. *IEEE Transactions on pattern analysis and machine intelligence*, **20**, 113-125.

8 Boudaoud, A., Burian, A., Borowska-Wykręt, D., Uyttewaal, M., Wrzalik, R., Kwiatkowska, D. and Hamant, O. (2014) FibrilTool, an ImageJ plug-in to quantify fibrillar structures in raw microscopy images. *Nat. Protocols*, **9**, 457-463.

9 Degasperi, A., Birtwistle, M.R., Volinsky, N., Rauch, J., Kolch, W. and Kholodenko, B.N. (2014) Evaluating strategies to normalise biological replicates of Western blot data. *PloS one*, **9**, e87293.

10 D'Agostino, R.B. and Belanger, A. (1990) A Suggestion for Using Powerful and Informative Tests of Normality. *The American Statistician*, **44**, 316-321.
